# Exploring the accuracy of the Xpert MTB/RIF assay in detecting lymph node tuberculosis: A systematic review and meta-analysis

**DOI:** 10.1371/journal.pone.0321507

**Published:** 2025-05-07

**Authors:** Zi-Ke Chen, Zhao Wu, Ting Ye, Chao-Yang Lin, Xu-Guang Guo

**Affiliations:** 1 Department of Clinical Laboratory Medicine, Guangdong Provincial Key Laboratory of Major Obstetric Diseases, Guangdong Provincial Clinical Research Center for Obstetrics and Gynecology, The Third Affiliated Hospital of Guangzhou Medical University, Guangzhou, China; 2 Department of Clinical Medicine, The Third Clinical School of Guangzhou Medical University, Guangzhou, China; 3 Guangzhou Key Laboratory for Clinical Rapid Diagnosis and Early Warning of Infectious Diseases, King Med School of Laboratory Medicine, Guangzhou Medical University, Guangzhou, China; The University of Georgia, UNITED STATES OF AMERICA

## Abstract

**Background:**

The Xpert MTB/RIF assay has exhibited high diagnostic efficiency in detecting lymph node tuberculosis (LNTB). However, different gold standards and types of LNTB specimens may impact Xpert’s diagnostic accuracy. This meta-analysis compared the performance of Xpert MTB/RIF on fine needle aspiration (FNA) and tissue samples against that of culture and composite reference standards (CRS) for LNTB diagnosis. In addition, we further reported the diagnostic accuracy of the Xpert MTB/RIF assay in identifying LNTB in different age groups and rifampicin resistance in LNTB patients.

**Methods:**

A systematic search of Embase, Cochrane Library, PubMed, Web of Science, and Scopus up to October 26, 2023, was conducted. Studies comparing Xpert MTB/RIF with culture and CRS were selected. Meta-analyses and meta-regression were performed using Stata and Meta Disc software.

**Results:**

In 9 studies, Xpert MTB/RIF was compared with CRS, while in 24 studies, it was compared with culture. The sensitivity and specificity of the Xpert MTB/RIF test were 85% and 97%, respectively, against CRS and 85% and 78%, respectively, against culture. FNA sensitivity and specificity were 93% and 88% respectively against CRS and 87% and 77% respectively against culture. For tissue samples, the sensitivity and specificity were 74% and 100%, respectively, against CRS and 74% and 77%, respectively, against culture. For the adult cohort (>14 years), the sensitivity and specificity of FNA samples were 80% and 75% against culture and 85% and 88% respectively against CRS. The pooled sensitivity and specificity of Xpert for detecting rifampicin resistance were 90% and 99% respectively.

**Conclusion:**

Xpert MTB/RIF demonstrated excellent accuracy for diagnosing LNTB and rifampicin resistance, with FNA samples outperforming tissue samples acquired through biopsy and CRS as superior to culture standards.

## Introduction

As the most common form of extrapulmonary tuberculosis (EPTB), lymph node tuberculosis (LNTB) exhibits swollen lymph node, fever, night sweat, representing approximately 35% of EPTB cases in developing countries [1]. Without a quick and accurate diagnostic method, treating and controlling LNTB poses a significant challenge [2]. Currently, culture remains the gold standard for diagnosis. Nonetheless, an extended turnaround time of 6–8 weeks and relatively low sensitivity pose challenges [3]. Similarly, acid-fast bacillus (AFB) smears have low sensitivity. The presence of granuloma and caseous necrosis in tissue histopathology supported the diagnosis of LNTB. However, these features are not specific, as they can be found in other diseases [4].

Xpert MTB/RIF is capable of detecting Mycobacterium tuberculosis complex (MTBC) DNA within a mere 2 hours [5]. This assay has been demonstrated to exhibit high diagnostic accuracy, specifically in detecting LNTB [6]. The World Health Organization (WHO) has provisionally recommended using Xpert MTB/RIF for testing lymph node samples in individuals with suspected EPTB. This recommendation was supported by limited and highly heterogeneous data, and underscored the need for further research to evaluate the performance of Xpert in EPTB patients, particularly in the LNTB [7]. Although Xpert Ultra exhibits greater sensitivity, have more molecular targets, and is able to detect rifampicin resistance more effectively [8], it lacks sufficient original articles and data. By contrast, Xpert MTB/RIF is used to detect various sample types of tuberculosis (TB) in numerous studies and exhibits greater specificity than Xpert Ultra [9–11]. In addition, the diagnostic accuracy of Xpert MTB/RIF has been continuously reported in newly published articles [12–14], indicating its high clinical significance.

A consensus has not been reached on the optimal specimens for patients suspected of LNTB [15]. Different sample processing procedures may also affect the interpretation of the results [6]. While FNA is an easy-to-perform and quick diagnostic technique, it exhibits variable sensitivity, potentially explained by the limited quantity of bacilli in FNA samples [16]. Pathological diagnosis is highly sensitive when utilizing the most invasive biopsy specimens [15]. Furthermore, we implemented a composite reference standard (CRS). CRS consists of results including histopathological examination, smears, results of biochemical tests, clinical manifestations, radiological findings, culture and/or response to antituberculosis therapy, serving as a benchmark in addition to culture for TB classification [17]. CRS classifies the presence of TB as positive if at least one component test is positive.

TB in children is typically paucibacillary and young children cannot voluntarily produce sputum specimens, making the microbiological confirmation of TB more difficult in children [18]. WHO found children younger than 14 years old gained poorer access to diagnosis and treatment. 16% of death cases from TB were children while children cases shared only 11% of whole age groups [19]. Hence, this meta-analysis was implemented to assess the diagnostic accuracy of the Xpert MTB/RIF assay for detecting various specimen types (including FNA and tissue samples) in individuals in different age groups suspected of LNTB compared to both CRS and mycobacterial culture methods.

## Materials and methods

### Study protocol

This meta-analysis was conducted in accordance with the guidelines of the Preferred Reporting Items for Systematic Reviews and Meta-Analyses (PRISMA)(S1 File) (registration number: CRD42024501482).

### Search Strategy

A comprehensive and systematic search was carried out using the PubMed, Cochrane Library, Embase, Web of Science, and Scopus databases, covering studies published before October 26, 2023. The search formula ((Tuberculosis, Lymph Node[Mesh]) AND (Xpert MTB/RIF[All Fields])) was applied to PubMed without restrictions. Analogous formulas were used for the Cochrane Library, Embase, Web of Science, and Scopus databases. Additionally, manual searches of review references and included articles were conducted to identify further relevant studies. The search did not impose restrictions on the publication year or geographic location. The relevant literature was screened based on the inclusion and exclusion criteria. Ultimately, the meta-analysis encompassed articles that met the designated criteria.

### Study selection

The inclusion criteria were as follows: (1) English-language literature, (2) literature providing data for a fourfold table (including true positive (TP), true negative (TN), false positive (FP), and false negative (FN) values), (3) a full-text original study assessing the accuracy of Xpert in diagnosing LNTB, (4) specimens obtained from humans, and (5) a gold standard defined as culture or CRS for LNTB diagnosis.

The exclusion criteria included various aspects: (1) absence of Xpert instrument utilization; (2) inclusion of meta-analyses, conference abstracts, case reports, pathology reports, and editorials; (3) studies with a sample size less than ten; (4) inconsistencies between the disease being studied and the intended use of the test for LNTB; (5) lack of access to tetrad data; and (6) absence of a gold standard.

### Reference standard

Different reference standards may impact diagnostic validity [20]. Given the limited presence of mycobacteria in EPTB, using culture as a reference standard could result in an inaccurate estimation of the true specificity of Xpert MTB/RIF. CRS might itself have lower specificity (more non-TB cases could be classified as TB cases), potentially leading to false negative results with Xpert MTB/RIF and thus underestimating its actual sensitivity [21,22]. Hence, conducting a study that compares Xpert MTB/RIF to both conventional culture and CRS could yield a more reliable spectrum of sensitivity and specificity.

### Literature screening and data collection

Two panelists independently evaluated candidate articles by scrutinizing titles, abstracts, and full texts for inclusion and independently extracted necessary information from each article, with cross-checking. The data extracted included author, year, country, TP, FP, FN, and TN values; reference standards; reference type; and other parameters. Missing data were not included in the analysis. Discrepancies between the two datasets were resolved through discussion with a third panel. Two sets of data, each with different reference criteria, were processed.

### Assessment of study quality

Two investigators independently classified studies according to two reference standards (CRS and culture) and conducted separate quality assessments employing the revised Quality Assessment of Diagnostic Accuracy Studies (QUADAS-2) tool (S2 File). Assessment of publication bias cannot be conducted owing to the inapplicability of these methods to diagnostic accuracy studies.

### Data synthesis and statistical analysis

In each study, values for TP, FP, FN, and TN were collected. Using bivariate random-effects models, forest plots were generated to depict the sensitivity and specificity of Xpert MTB/RIF with 95% CIs against CRS or culture. The area under the summary receiver operating characteristic (SROC) curve was determined. Heterogeneity between studies and reference standards was assessed using I2 statistics, where values exceeding 50% were deemed to indicate significant heterogeneity [23,24].

Potential sources of heterogeneity, including different sample types, sample conditions, decontamination techniques, and homogenization methods, were investigated through subgroup and meta-regression analyses. For meta-analysis, a minimum of four published studies per predefined variable type were necessary. Comparisons of the CRS and culture data were performed. Meta-DiSc 1.4 software was used to analyze the data extracted from the included studies and to plot the sroc curves. Forest plots illustrating sensitivity and specificity with 95% confidence intervals for each study were generated using Stata version 15.0 (Stata Corp, College Station, TX) and midas command packages.

## Results

### Study evaluation

**Fig 1:**
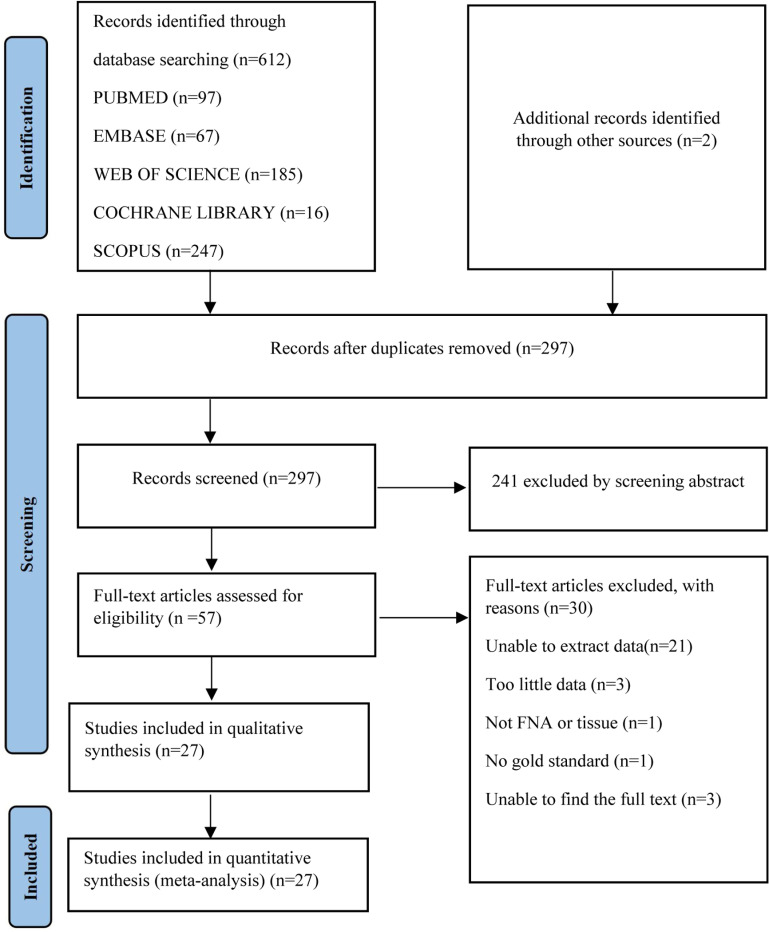
Literature retrieval flow chart. A total of 97, 67, 185, 16 and 247 articles were found from PUBMED, EMBASE, WEB OF SCIENCE, COCHRANE LIBRARY and SCOPUS respectively.

In this meta-analysis, we identified 614 candidate articles from relevant databases and 2 additional articles from other sources. Among these, 97 articles were from PubMed, 67 from Embase, 16 from the Cochrane Library, 185 from the Web of Science, and 247 from Scopus. Of the 614 articles, 317 were identified as duplicates. After reviewing the titles and abstracts, 57 articles were subjected to the full-text screening process. After excluding 21 articles due to inability to extract data, 3 articles with insufficient data, 1 article with inappropriate sample type, 1 article not reporting a gold standard, and 3 articles where the fulll text could not be found, 27 articles were ultimately included for full-text review and meta-analysis. Each article evaluated specimens ranging from 11 to 379, with a median of 82. All studies were conducted in English. The screening process is illustrated in Fig 1.

If an article utilized two distinct standards within the same research, we classified it as comprising two separate studies. According to these guidelines, 33 distinct studies were included: 9 assessed Xpert MTB/RIF against CRS, and 24 evaluated Xpert MTB/RIF against culture (Table 1). Among these, 22 studies employed FNA samples, while 11 utilized biopsy tissue sample

**Table 1:** Characteristics of included articles.

Author’s Name	Year	Country	Source of Sample	Gold Standard	Patient selection method	Sample Size	TP	FP	FN	TN	Decontaminate method	Sample condition	Location	Homogenisation	Sample ratio
A. Van Rie	2013	South Africa	clinical specimen	Culture	Consecutive	344	139	23	10	172	No	Fresh	Peripheral	No	2:01
Abebe G	2015	Southwest Ethiopia	clinical specimen	Culture	Consecutive	136	76	11	7	42	NALC-NaOH	Frozen	Peripheral	No	3:01
Ablanedo TY	2014	Mexico	clinical specimen	Culture	Consecutive	68	15	0	0	53	NALC-NaOH	Frozen	Peripheral	No	2:01
Asnake Simieneh.a	2022	Ethiopia	clinical specimen	Culture	Consecutive	147	16	0	7	124	NALC-NaOH	Fresh	Peripheral	No	2:01
Asnake Simieneh.b	2022	Ethiopia	clinical specimen	CRS	Consecutive	19	12	0	2	5	NALC-NaOH	Fresh	Peripheral	No	2:01
Atnafu A	2022	Ethiopia	clinical specimen	Culture	Consecutive	96	33	10	3	50	NALC-NaOH	Fresh	Peripheral	No	3:01
Bholla M	2016	Tanzania	clinical specimen	CRS	Consecutive	62	11	3	8	40	No	Fresh	Peripheral	No	3:01
Biadglegne,F	2013	Ethiopia	clinical specimen	Culture	Convenience	220	29	56	3	132	NALC-NaOH	Frozen	Peripheral	No	3:01
CHE Nan Ying	2017	China	clinical specimen	Culture	Consecutive	82	22	47	0	13	No	Fresh	Peripheral	Mechanical	2:01
Coetzzee L	2014	South Africa	clinical specimen	Culture	Consecutive	72	21	13	4	34	No	Fresh	Peripheral	No	2:01
Dhasmana DJ	2014	United Kingdom	clinical specimen	CRS	Consecutive	116	24	3	12	77	No	Fresh	Mediastinal	No	Unknow
Huang S.a	2020	China	clinical specimen	CRS	Consecutive	62	43	9	0	10	NALC-NaOH	Fresh	Peripheral	Mechanical	2:01
Huang S.b	2020	China	clinical specimen	Culture	Consecutive	53	15	0	28	10	NALC-NaOH	Fresh	Peripheral	Mechanical	2:01
Jitendra Singh Rathour	2019	India	clinical specimen	Culture	Consecutive	27	4	3	1	19	No	Fresh	Peripheral	No	2:01
Ligthelm,L.J.a	2011	South Africa	clinical specimen	CRS	Consecutive	48	29	2	1	16	No	Fresh	Peripheral	No	2:01
Ligthelm,L.J.b	2011	South Africa	clinical specimen	Culture	Consecutive	48	28	3	1	16	No	Fresh	Peripheral	No	2:01
Liwei Yao	2022	China	clinical specimen	CRS	Consecutive	114	72	0	28	14	No	Fresh	Peripheral	No	2:01
Minnies S	2021	South Africa	clinical specimen	Culture	Consecutive	94	7	33	9	45	No	Unknow	Peripheral	No	2:01
Mohan V	2021	India	clinical specimen	Culture	Consecutive	60	16	14	2	28	No	Fresh	Peripheral	No	Unknow
Mukhinda S	2022	India	clinical specimen	Culture	Consecutive	145	36	53	6	50	NALC-NaOH	Fresh	Peripheral	Mechanical	2:01
N. Sharif	2023	Pakistan	clinical specimen	Culture	Consecutive	111	48	26	32	5	NALC-NaOH	Sample condition	Peripheral	No	2:01
Rebecca B	2018	South India	clinical specimen	Culture	Consecutive	22	12	9	1	0	No	Frozen	Peripheral	No	Unknow
Sarfaraz S	2018	Pakistan	clinical specimen	Culture	Consecutive	261	44	38	23	156	NALC-NaOH	Fresh	Peripheral	No	Unknow
Sushil Pandey.a	2017	Australia	clinical specimen	Culture	Consecutive	14	9	1	1	3	NALC-NaOH	Fresh	Peripheral	No	2:01
Sushil Pandey.b	2017	Australia	clinical specimen	Culture	Consecutive	11	5	2	4	0	NALC-NaOH	Fresh	Peripheral	No	2:01
Suzana, S.a	2016	India	clinical specimen	Culture	Consecutive	66	19	19	1	27	No	Fresh	Peripheral	No	2:01
Suzana, S.b	2016	India	clinical specimen	CRS	Consecutive	67	38	0	18	11	No	Fresh	Peripheral	No	2:01
Tahseen S	2022	Pakistan	clinical specimen	Culture	Consecutive	379	83	41	50	205	No	Fresh	Peripheral	Mechanical	Unknow
Teyim Pride Mbuh	2019	Cameroon	clinical specimen	Culture	Consecutive	46	24	4	3	15	NALC-NaOH	Fresh	Peripheral	Mechanical	2:01
Tortoli, E.a	2012	Italy	clinical specimen	Culture	Consecutive	118	24	4	5	85	NALC-NaOH	Frosen	Peripheral	No	2:01
Tortoli, E.b	2012	Italy	clinical specimen	CRS	Consecutive	119	28	1	5	85	NALC-NaOH	Frosen	Peripheral	No	2:01
Wenwen Sun	2021	China	clinical specimen	CRS	Consecutive	307	237	0	35	35	No	Fresh	Peripheral	No	2:01
Willy Ssengooba	2020	Uganda	clinical specimen	Culture	Consecutive	353	14	17	2	320	NALC-NaOH	Fresh	Peripheral	Mechanical	2:01

### Study quality

**Fig 2:**
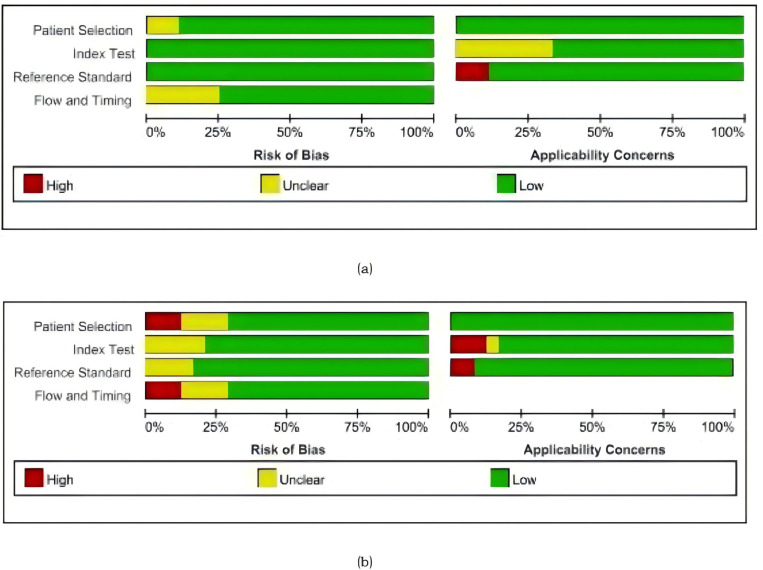
Methodological quality graphs (risk of bias and applicability concerns) as percentages across the included studies. **(a)** Composite reference standard. **(b)** Culture reference standard.

A quality assessment of diagnostic accuracy studies against CRS or culture as a reference standard was carried out using QUADAS-2 (Fig 2).

### Diagnostic accuracy of the Xpert MTB/RIF Assay for identifying LNTB

**Fig 3:**
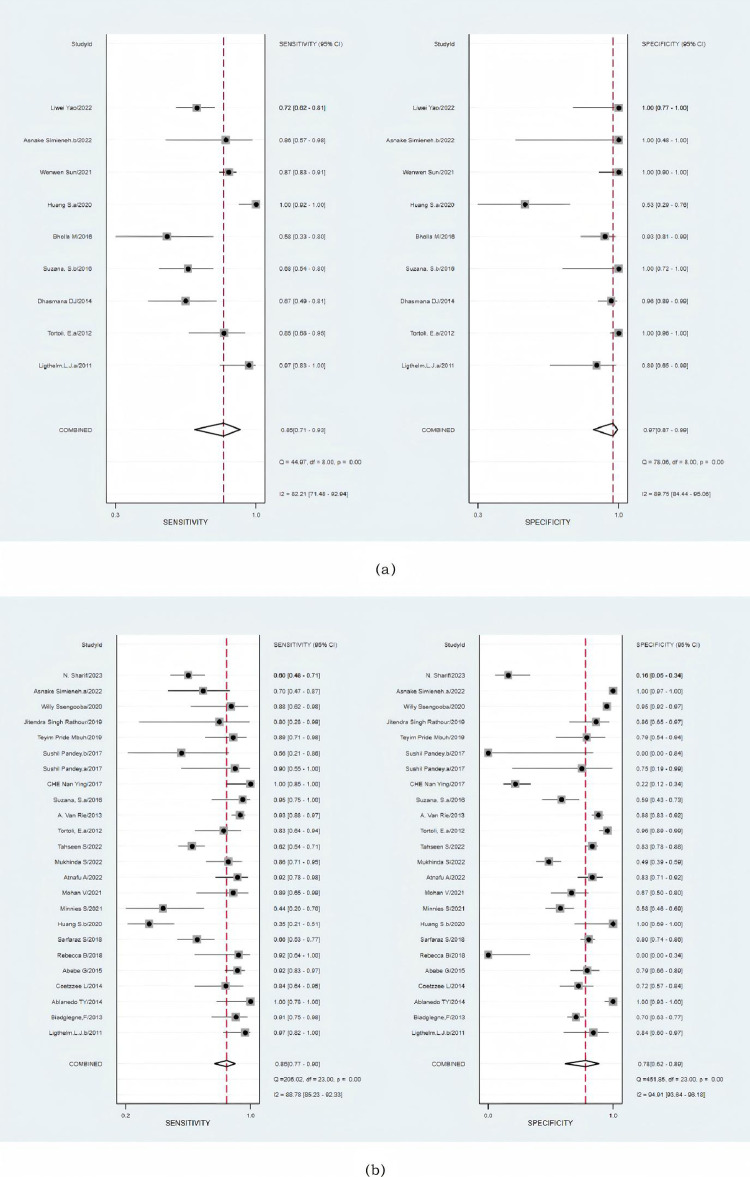
(a) Forest plot of the sensitivity and specificity of Xpert for tuberculosis detection compared with a composite reference standard. (b) Forest plot of the sensitivity and specificity of Xpert for tuberculosis detection compared with the culture reference standard. The squares represent the sensitivity and specificity of a study, and the black line represents their confidence intervals. The diamonds represent the pooled sensitivity and specificity and their confidence intervals.

In nine studies, a total of 914 FNAs or tissue samples were compared with those from patients with CRS. The combined sensitivity of the Xpert MTB/RIF assay was 85% (95% CI: 71–93%; I2: 82%), while the aggregated specificity of the Xpert MTB/RIF assay was 97% (95% CI: 87–99%; I2: 90%) (Fig 3(a)). Notably, significant heterogeneity was observed in both sensitivity and specificity. The estimated area under the curve (AUC) against CRS, the gold standard in the Xpert assay, was 0.97 (95% CI: 9598%) (S1 Fig). Compared with culture, the overall sensitivity of Xpert MTB/RIF was 85% (95% CI: 77–90%; I2:89%), and the specificity was 78% (95% CI: 62–89%; I2: 95%), with 2973 FNAs or tissue specimens from 24 studies (Fig 3(b)), indicating significant heterogeneity. The AUC of the SROC was 0.89 (95% CI: 86–91%) compared with that of culture, indicating excellent overall diagnostic validity (S2 Fig).

**Fig 4:**
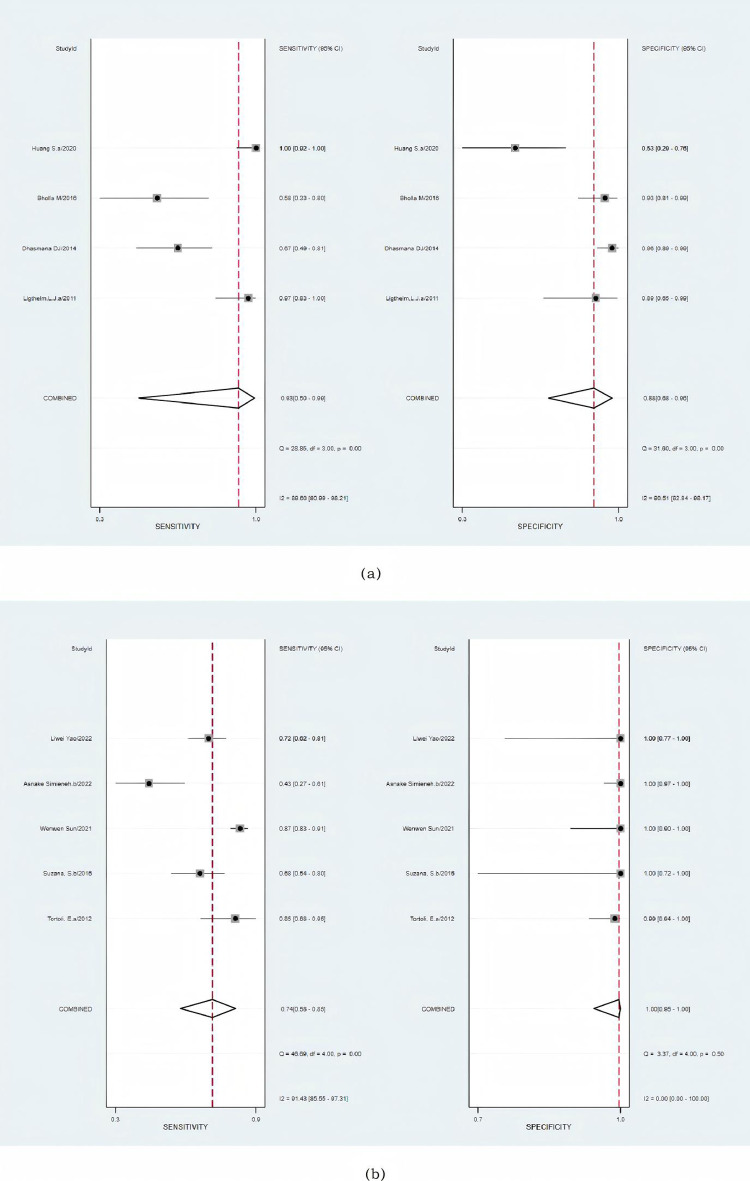
Forest plot of the sensitivity and specificity of Xpert for detecting tuberculosis versus a composite reference standard. **(a)** FNA samples. **(b)** Tissue samples. The squares represent the sensitivity and specificity of a study, and the black line represents their confidence intervals. The diamonds represent the pooled sensitivity and specificity and their confidence intervals.

The combined sensitivity of Xpert MTB/RIF for FNA specimens compared with CRS specimens was 93% (95% CI: 50–99%; I2: 90%), while its specificity was 88% (95% CI: 68–96%; I2: 91%) (Fig 4(a)). The AUC of the SROC was 0.95 (95% CI: 93–97%), indicating excellent diagnostic accuracy (S3 Fig). The sensitivity of tissue specimens in the Xpert evaluation versus CRS was 74% (95% CI: 58–85%; I2: 91%), while its specificity was 100% (95% CI: 95–100%; I2 = 0) (Fig 4(b)). Furthermore, the estimated AUC of tissue specimens compared with that of CRS specimens in the Xpert trial was 0.95 (95% CI: 93–97%) (S4 Fig).

**Fig 5:**
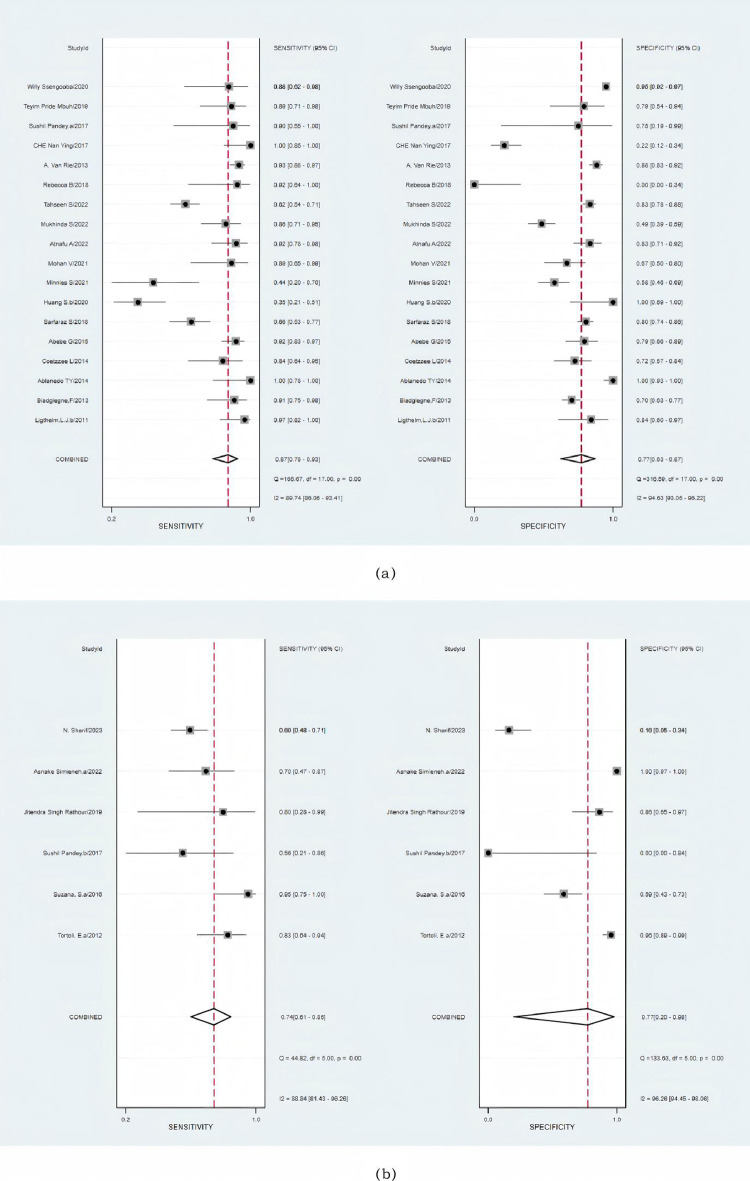
Forest plot of the sensitivity and specificity of the Xpert for tuberculosis detection versus culture. **(a)** FNA samples. **(b)** Tissue samples. The squares represent the sensitivity and specificity of a study, and the black line represents their confidence intervals. The diamonds represent the pooled sensitivity and specificity and their confidence intervals.

The aggregated sensitivity for Xpert MTB/RIF using FNA samples against culture was 87% (95% CI: 78–93%; I2: 90%), while the combined specificity was 77% (95% CI: 63–87%; I2: 95%) (Fig 5(a)). The estimated AUC of FNA samples against culture in the Xpert application was 0.90 (95% CI: 87–92%) (S5 Fig). The combined sensitivity and specificity of the Xpert MTB/RIF assay utilizing tissue samples compared to culture were 74% (95% CI: 61–85%; I2: 89%) and 77% (95% CI: 20–98%; I2 = 96%), respectively (Fig 5(b)). Notably, in contrast to culture, there was significant heterogeneity observed among the tissue samples. The estimated AUC of tissue samples against culture in the Xpert application was 0.78 (95% CI: 74–81%) (S6 Fig).

Meta-regression analysis revealed that compared with CRS, studies with FNA samples and tissue samples showed different but not statistically significant sensitivity (93% versus 74%, meta-regression P = 0.42) (S7 Fig). Comparative analysis revealed that studies utilizing FNA samples exhibited a lower specificity (88%) in contrast to those employing tissue samples (100%) compared to those employing CRS samples, with statistical significance noted (meta-regression P=0.04) (S8 Fig). While the sensitivity of studies utilizing FNA samples surpassed that of those employing tissue samples in comparison to culture (87% versus 74%), the disparity lacked statistical significance (meta-regression P = 0.29) (S9 Fig). Studies utilizing FNA samples and tissue samples showed the same specificity compared with culture(77% versus 77%), with no significant difference found (meta-regression P = 0.05) (S10 Fig).

Through subgroup and meta-regression analyses, we investigated the heterogeneity among studies by examining predefined subgroups related to decontamination methods (with or without NALC-NaOH), homogenization techniques (mechanical or alternative), and sample conditions (fresh or frozen). Meta-regression analysis revealed that none of these factors had an impact on the diagnostic performance of Xpert MTB/RIF compared to CRS and culture (meta-regression P > 0.01) (S11-13 Fig). Consequently, these variables were not attributed to the heterogeneity observed across the studies.

### Diagnostic accuracy of the Xpert MTB/RIF assay for identifying LNTB in different age groups

We delineated the population into two distinct age brackets, those aged 14 years and younger and those older than 14 years, to accurately reflect the pediatric and adult populations, respectively.

In our analysis, we constructed forest plots to evaluate the diagnostic performance of Xpert MTB/RIF across these age groups, utilizing both sample types and established gold standards for comparison. For the pediatric subset (≤14 years), FNA samples demonstrated a sensitivity and specificity of 87% (95% CI: 75–95%; I2:0%) and 90% (95% CI: 87–93%; I2:97%) against culture, respectively (S14-S15 Figs). However, the scarcity of literature, specifically, the presence of only a single study addressing tissue samples with both CRS and culture as gold standards and of studies addressing FNA samples with CRS as the gold standard, necessitates the acquisition of further original research to facilitate a comprehensive meta-analysis in this domain.

For the adult cohort (>14 years), the sensitivity and specificity of FNA samples, with culture as the gold standard, were 80% (95% CI: 76–84%; I2:92%) and 75% (95% CI: 72–79%; I2:97%), respectively (S16-S17 Figs). Similarly, FNA samples with CRS as the gold standard exhibited a sensitivity and specificity of 85% (95% CI: 75–92%; I2:95%) and 88% (95% CI: 80–94%; I2:95%), respectively (S18-19 Figs). The limited availability of studies, particularly the singular paper addressing tissue samples with CRS and culture as gold standards, precludes us from synthesizing the data for a more robust statistical analysis.

### Diagnostic accuracy of the Xpert MTB/RIF Assay for detecting rifampicin resistance in LNTB patients

Five studies reported the diagnostic performance of Xpert for rifampicin resistance. The pooled sensitivity and specificity were 90% (95% CI: 55%-100%) and 99% (95% CI: 96%-100%), respectively (S20-S21 Figs). Furthermore, the estimated AUC of rifampicin resistance in the Xpert application was 0.89 (95% CI: 93–97%) (S22 Fig).

## Discussion

Systematic reviews and meta-analyses published from 2014 to 2019 revealed the excellent diagnostic performance of Xpert MTB/RIF for LNTB [6,25–27]. However, no consensus has been reached on the diagnostic performance of the test to specimens obtained by different methods, including FNA and biopsy specimens, compared with different reference standards.

Similar studies have reported the diagnostic accuracy of Xpert MTB/RIF for different sample types and different reference criteria [26,28]. However, Kohli Mikashmi et al. lack adequate data on lymph node biopsy specimens examined by Xpert MTB/RIF and lack sufficient discussion, which decreases the reliability of the results [28]. Yu, Guocan, et al. were limited by various biases and remarkable heterogeneity [26]. Therefore, our research updated the articles and data included to assess the effectiveness of Xpert MTB/RIF in both biopsy and FNA specimens compared with different reference standards, offering new insights into specimens and reference standards selection for diagnosing LNTB.

Our research revealed that the overall sensitivity of Xpert MTB/RIF employing CRS as a reference standard in LNTB patients was 85%, while its specificity was 97%. Compared with the 85% sensitivity and 78% specificity of Xpert versus culture, employing CRS as a reference standard improved diagnostic performance of Xpert in our study. Similarly, a meta-analysis by Denkinger et al. reported a sensitivity of 81.2% and a specificity of 99.1% for detecting TB with Xpert MTB/RIF versus CRS, respectively. Moreover, it indicated 83.1% sensitivity and 93.6% specificity versus culture for lymph node tissues or FNA samples [6]. The specificity improved when the CRS was used as the gold standard in comparison with culture as a reference standard. CRS is a combination of component tests. Compared with a single imperfect reference standard, CRS combining multiple tests can be transparent and reproducible to reach the final diagnosis of TB [22]. However, subjectivity might be introduced in this process [22]. Samples were categorized as positive if any component test indicated TB. If CRS included more component tests, more TB specimens could be classified as positive and more non-TB samples could be diagnosed as TB wrongly, potentially leading to more false negatives and fewer false positives with Xpert MTB/RIF. With more tests added into CRS, the gain in sensitivity or specificity of CRS decreases and the clinical interpretability may diminish [22]. There is always a trade-off between sensitivity and specificity when combining various component tests [29]. And the diagnostic performance of Xpert could be biased when not all samples receive the CRS including same component tests [30]. To define a standardized and robust CRS in further investigation, complementary component tests that catch each other’s mistakes in detecting TB can be incorporated into CRS by assigning weights to reach the final diagnosis [22]. The reduced specificity observed in lymph node specimens compared to culture methods can be attributed to potential factors such as inadequate collection of specimens [28,31] and the utilization of decontaminated samples for culture inoculation. The latter involves a digestion and purification process using the N-acetyl-L-cysteine and sodium hydroxide method (NALC/NaOH), which may lead to the loss of viable mycobacteria and, consequently, false-negative culture results, particularly in paucibacillary specimens [32,33]. While our results aligned with this study, we observed significant heterogeneity among the studies. This may be explained by multiple reasons. First, the processing of lymph node specimens exhibited significant variability across and within studies, encompassing differences in sample purification with NALC-NaOH and machine homogenization [33]. This may have contributed to high heterogeneous data. Second, LNTB can infect lymph nodes in different parts of the body. Some included studies collected specimens solely in cervical lymph nodes, whereas other studies collected specimens in various lymph node sites such as cervical, axillary, and inguinal [34,35], potentially leading to high heterogeneity among the studies. Third, some studies exclusively included children or HIV-infected patients, possibly introducing some bias in our results [36].

Through meta-regression analysis, the sensitivity of Xpert MTB/RIF for FNA specimens was found to exceed that for tissue samples (93% versus 74% compared with CRS; 87% versus 77% compared with culture). However, this difference was statistically insignificant, regardless of the gold standard. This may be partly explained by the sample reagent buffer may not sufficiently homogenize the tissues, causing a potential decrease on sensitivity [37], while FNA specimens are more likely to be homogenized by contrast. Furthermore, tissue samples obtained through biopsy may contain a mixture of cellular and noncellular components, potentially diluting the concentration of mycobacteria. In contrast, more targeted FNA specimens reduce the dilution effect, making it easier to identify tuberculosis-specific markers [38]. The specificity of Xpert MTB/RIF for FNA specimens was lower than that for tissue specimens (88% versus 100% compared with CRS; 77% versus 77% compared with culture). Overall, the diagnostic accuracy of FNA exceeded that of tissue specimen methods in our study. As a first line diagnostic procedure, FNA is a simple, cost effective and highly accurate method in diagnosing TB. However, due to the limited number of bacilli in the FNA sample and the possibility to miss out extracting tissue in the lesion site, some samples may not be representative in the case of TB lesion, yielding false negatives on Xpert [16]. Repeat aspiration and using ultrasound-guided FNA may improve the quality of FNA and the detection rate of TB with Xpert [39,40].

Nonetheless, as a type of LNTB, mediastinal tuberculous lymphadenitis, endosonography is the recommended technique for diagnosing mediastinal lymphadenopathies. Mondoni, Michele, et al. reported a lower sensitivity (61%) of Xpert MTB/RIF in the diagnosis of mediastinal TB lymphadenitis obtained by endoscopic ultrasound-guided needle aspiration techniques than that reported for FNA samples in our study (93% compared with CRS, 87% compared with culture) and a higher specificity of endosonography compared with our study (89% versus 87% against CRS; 89% versus 77% against culture) [41]. Similarly, Chhajed, Prashant N et al. reported 77% sensitivity of Xpert in diagnosing mediastinal tuberculous lymphadenitis through endosonography [42]. Kohli, Mikashmi et al. reported 88.9% sensitivity and 86.2% specificity of Xpert in diagnosing LNTB [28]. The discrepancy in diagnostic performance may be due to the following reasons. First, mediastinal lymph nodes are more difficult to sample than peripheral lymph nodes and have a low bacillary load. Second, the decontamination procedure or the existence of a caseous necrotic lesion may lead to negative culture results and false-positive Xpert results, lowering the sensitivity of Xpert since such false-positive results could be true-positivity [42]. In addition, if the concentration of mycobacteria is below the detection threshold of Xpert MTB/RIF, the diagnosis could result in Xpert MTB/RIF-negative but TB culture-positive results, decreasing the sensitivity [16]. Third, the indications for mediastinal tuberculous lymphadenitis at the site of aspiration were made only by the pulmonology staff performing endosonography, which can be subjective, and quite a few individuals had enlarged intrathoracic lymph nodes due to a history of malignant diseases, which may have led to incorrect and unnecessary aspiration [43].

According to our study, a multistep approach for managing suspected LNTB is recommended: less invasive FNA, followed by comprehensive examinations such as Xpert MTB/RIF and pathological tests, to enhance diagnostic sensitivity. If the first step yields a negative result, a more invasive technique (such as biopsy) is recommended. Pathological examination, rather than Xpert MTB/RIF, may be employed for further validation, as the assay did not enhance sensitivity when applied to biopsy-obtained samples.

Due to the limited presence of mycobacteria, CRS may be more suitable for detecting LNTB. However, the composition of the CRS differed among the studies analyzed in this research. One potential cause of variability among studies could stem from this factor. Variability existed in the processing procedure of lymph node specimens among studies, encompassing sample conditions, decontamination, and homogenization. Nonetheless, meta-regression analysis did not reveal that the variability markedly impacted the outcomes or contributed to heterogeneity.

We conducted a subgroup analysis of the child and adult populations. The diagnostic Xpert of the child group with FNA samples showed 87% sensitivity and 90% specificity. A review from the WHO reported 86% sensitivity and 81% specificity of Xpert MTB/RIF compared with culture for detecting peripheral lymph node TB in children, with high heterogeneity indicated [44]. The diagnostic Xpert of the adult group with FNA samples showed 80% sensitivity and 75% specificity with culture as the gold standard and 85% sensitivity and 88% specificity with CRS. Similarly, Kohli, Mikashmi, et al. reported 88.9% sensitivity and 86.2% specificity of Xpert MTB/RIF in lymph node aspirates compared with culture as a reference standard, and 81.6% sensitivity and 96.4% specificity against CRS were reported for adults [28]. As LNTB has different epidemiological characteristics in children and adults, we hope that this subgroup analysis of different age ranges can provide a reference and attract more attention to this issue.

Our study suggested that, as a rapid diagnostic tool, Xpert MTB/RIF has excellent performance in detecting rifampicin resistance in LNTB patients, with a pooled sensitivity and specificity of 90% and 99%, respectively. Similarly, a study from Liu, Aimei et al. reported a pooled sensitivity and specificity of 83.8% and 100%, respectively, for detecting rifampicin resistance in suspected suspected drug-resistant TB [45]. Kohli, Mikashmi et al. reported 96.5% sensitivity and 99.1% specificity of Xpert MTB/RIF in detecting rifampicin resistance in extrapulmonary specimens [28]. However, our results are based on only 5 studies and should be interpreted with caution.

Our meta-analysis has limitations. First, we acknowledge the possibility of missing studies despite a thorough search, some of which did not differentiate specimen types. Second, some included studies focused solely on one sample type, whereas one study [33] collected multiple specimens, potentially introducing bias to our results. Besides, our finding is unable to offer more clues about the main strains of LNTB since Xpert MTB/RIF assay can’t distinguish causative agents of TB lymphadenitis between M. tuberculosis, M. bovis or M. orygis [32]. Furthermore, CRS varied among studies. Typically, studies have employed a CRS involving culture, microscopy, or cytology of FNA samples [46,47]. Meanwhile, some studies reported clinical symptoms or responses to anti-TB treatment, which might bias our final research data. In addition, some included studies exclusively included children, whereas others included all age groups or specifically enrolled adults, possibly introducing some bias in our results.

Furthermore, some included studies specifically focused on HIV-infected patients, and certain studies utilized the response to anti-tuberculosis therapy as a standard to screen the target population [48]. Patients in the study were typically referred to the research center from primary health care clinics and presented with more severe cases [48], potentially introducing selection bias. Notably, significant heterogeneity existed among the studies, requiring cautious interpretation of the pooled estimates.

## Conclusion

The meta-analysis revealed that in comparison to CRS, Xpert MTB/RIF exhibited a sensitivity of 85% and specificity of 97%. Conversely, compared to culture, the sensitivity and specificity were 85% and 78%, respectively. FNA samples demonstrated a sensitivity of 93% and specificity of 88% against CRS and 87% and 77% against culture, respectively. The tissue samples displayed a sensitivity of 74% and specificity of 100% against CRS and 74% and 77% against culture, respectively. Overall, Xpert MTB/RIF shows significant diagnostic accuracy for LNTB, with FNA samples outperforming tissue samples acquired through biopsy.

## Supporting information

S1 Fig SROC curves using CRS as the gold standard are included in the articles. (ZIP)

S2 Fig Forest plots of SROC curves using Culture as the gold standard are included in articles. (ZIP)

S3 Fig SROC curve using FNA samples and CRS as the gold standard. (ZIP)

S4 Fig SROC curve using tissue samples and CRS as the gold standard. (ZIP)

S5 Fig SROC curve using FNA samples and culture as the gold standard. (ZIP)

S6 Fig SROC curve using tissue samples against culture as the gold standard. (ZIP)

S7 Fig Meta-regression analysis of the sensitivity of FNA samples and tissue samples using CRS as the gold standard. (ZIP)

S8 Fig Meta-regression analysis of specificity of FNA samples and tissue samples using CRS as the gold standard. (ZIP)

S9 Fig Meta-regression analysis of the sensitivity of FNA samples and tissue samples using Culture as the gold standard. (ZIP)

S10 Fig Meta-regression analysis of specificity of FNA samples and tissue samples using Culture as the gold standard. (ZIP)

S11 Fig Results of meta-regression analysis of sample ratio, contaminate method, homogenization and sample condition of FNA samples with CRS as the gold standard: (a) sample rate. (b) purification method. (c) homogenization. (d) sample condition.
(ZIP)

S12 Fig Results of the meta-regression analysis of tissue samples with CRS as the gold standard: (a) Purification methods. (b) Sample conditions.
(ZIP)

S13 Fig Results of the meta-regression analysis of the sample ratio, purification method, and homogenization of cultured FNA samples as the gold standard: (a) Sample ratio. (b) decontamination method. (c) Homogenization.
(ZIP)

S14 Fig Plot of specificity results for FNA samples from patients less than 14 years of age with culture as the gold standard. (ZIP)

S15 Fig Plot of specificity results for FNA samples from patients less than 14 years of age with culture as the gold standard. (ZIP)

S16 Fig Plot of sensitivity results for FNA samples from individuals over 14 years of age with culture as the gold standard. (ZIP)

S17 Fig Plot of specificity results for FNA samples from individuals over 14 years of age with culture as the gold standard. (ZIP)

S18 Fig Plot of sensitivity results for FNA samples from individuals over 14 years of age with CRS as the gold standard. (ZIP)

S19 Fig Plot of specificity results for FNA samples from individuals over 14 years of age with CRS as the gold standard. (ZIP)

S20 Fig Plot of sensitivity results for rifampicin resistance. (ZIP)

S21 Fig Plot of specificity results for rifampicin resistance. (ZIP)

S22 Fig SROC results for rifampicin resistance. (ZIP)

S1 File The PRISMA Checklist. (ZIP)

S2 File quality assessments employing the revised Quality Assessment of Diagnostic Accuracy Studies two(QUADAS-2).
(ZIP)

S3 File all data extracted from the primary research sources.
(ZIP)

S4 Fil Risk of bias for each study.
(ZIP)

S5 File All studies identified in the literature search. (ZIP)

S6 File Search strategy. (ZIP)

## Abbreviations

LNTB: lymph node tuberculosis
FNA: fine needle aspiration
CRS: composite reference standard
EPTB: extrapulmonary tuberculosis
AFB: acid-fast bacillus
MTBC: Mycobacterium tuberculosis complex
TB: tuberculosis
SROC: summary receiver operating characteristic
AUC: area under the curve
WHO: World Health Organization
TP: true positive
TN: true negative
FP: false positive
FN: false negative.

## References

[1] Handa U, Mundi I, Mohan S. Nodal tuberculosis revisited: a review. J Infect Dev Ctries. 2012;6(1):6–12. doi: 10.3855/jidc.2090 22240421

[2] W.H. Organization(WHO). Global Tuberculosis Report. 2019.

[3] Lewinsohn DM, Leonard MK, LoBue PA, Cohn DL, Daley CL, Desmond E, et al. Official American Thoracic Society/Infectious Diseases Society of America/Centers for Disease Control and Prevention Clinical Practice Guidelines: diagnosis of tuberculosis in adults and children. Clin Infect Dis. 2017;64(2):111–5. doi: 10.1093/cid/ciw778 28052967 PMC5504475

[4] Asano S. Granulomatous lymphadenitis. J Clin Exp Hematop. 2012;52(1):1–16. doi: 10.3960/jslrt.52.1 22706525

[5] Boehme CC, Nicol MP, Nabeta P, Michael JS, Gotuzzo E, Tahirli R, et al. Feasibility, diagnostic accuracy, and effectiveness of decentralised use of the Xpert MTB/RIF test for diagnosis of tuberculosis and multidrug resistance: a multicentre implementation study. Lancet. 2011;377(9776):1495–505. doi: 10.1016/S0140-6736(11)60438-8 21507477 PMC3085933

[6] Denkinger CM, Schumacher SG, Boehme CC, Dendukuri N, Pai M, Steingart KR. Xpert MTB/RIF assay for the diagnosis of extrapulmonary tuberculosis: a systematic review and meta-analysis. Eur Respir J. 2014;44(2):435–46. doi: 10.1183/09031936.00007814 24696113

[7] WHO Guidelines Approved by the Guidelines Review Committee. Xpert MTB/RIF implementation manual: technical and operational ‘How-To’; practical considerations. Geneva: World Health Organization Copyright © World Health Organization 2014; 2014.

[8] Yadav S, Rawal G, Jeyaraman M, Jeyaraman N. Advancements in tuberculosis diagnostics: a comprehensive review of the critical role and future prospects of Xpert MTB/RIF Ultra technology. Cureus. 2024;16:e57311. 38690500 10.7759/cureus.57311 PMC11059844

[9] Nasrin R, Uddin MKM, Kabir SN, Rahman T, Biswas S, Hossain A, et al. Xpert MTB/RIF ultra for the rapid diagnosis of extrapulmonary tuberculosis in a clinical setting of high tuberculosis prevalence country and interpretation of “trace” results. Tuberculosis (Edinb). 2024;145:102478. doi: 10.1016/j.tube.2024.102478 38218133

[10] Slail MJ, Booq RY, Al-Ahmad IH, Alharbi AA, Alharbi SF, Alotaibi MZ, et al. Evaluation of Xpert MTB/RIF ultra for the diagnosis of extrapulmonary tuberculosis: a retrospective analysis in Saudi Arabia. J Epidemiol Glob Health. 2023;13(4):782–93. doi: 10.1007/s44197-023-00150-z 37707714 PMC10686912

[11] Li M, Qiu Y, Guo M, Qu R, Tian F, Wang G, et al. Comparison of Xpert MTB/RIF ultra with Xpert MTB/RIF for the detection of Mycobacterium tuberculosis and rifampicin resistance in a primary-level clinic in rural China. Tuberculosis (Edinburgh, Scotland). 2023;142:102397. 37597313 10.1016/j.tube.2023.102397

[12] Li T-X, Wang J, Yang Y-S, Wang P-S, Zhou G, Liao C-Y, et al. Evaluation of Xpert MTB/RIF assay for the diagnosis of extrapulmonary tuberculosis in Southwest China. PLoS Negl Trop Dis. 2023;17(6):e0011403. doi: 10.1371/journal.pntd.0011403 37363913 PMC10328326

[13] Li W, Sha W. Diagnosis of chest wall tuberculosis using fine needle aspiration: a single-center experience. Infect Drug Resistance. 2023;16:2281–90. 10.2147/IDR.S404804 PMC10122463 37095781

[14] Atnafu A, Desta K, Girma S, Hailu D, Assefa G, Araya S, et al. Integration of cytopathology with molecular tests to improve the lab diagnosis for TBLN suspected patients. PLoS One. 2022;17(3):e0265499. doi: 10.1371/journal.pone.0265499 35358212 PMC8970391

[15] Salvador F, Los-Arcos I, Sánchez-Montalvá A, Tórtola T, Curran A, Villar A, et al. Epidemiology and diagnosis of tuberculous lymphadenitis in a tuberculosis low-burden country. Medicine (Baltimore). 2015;94(4):e509. doi: 10.1097/MD.0000000000000509 25634205 PMC4602977

[16] Tadesse M, Abebe G, Abdissa K, Aragaw D, Abdella K, Bekele A, et al. GeneXpert MTB/RIF assay for the diagnosis of tuberculous lymphadenitis on concentrated fine needle aspirates in high tuberculosis burden settings. PLoS One. 2015;10(9):e0137471. doi: 10.1371/journal.pone.0137471 26366871 PMC4569183

[17] Vadwai V, Boehme C, Nabeta P, Shetty A, Alland D, Rodrigues C. Xpert MTB/RIF: a new pillar in diagnosis of extrapulmonary tuberculosis?. J Clin Microbiol. 2011;49(7):2540–5. doi: 10.1128/JCM.02319-10 21593262 PMC3147857

[18] Kay AW, González Fernández L, Takwoingi Y, Eisenhut M, Detjen AK, Steingart KR, et al. Xpert MTB/RIF and Xpert MTB/RIF Ultra assays for active tuberculosis and rifampicin resistance in children. Cochrane Database Syst Rev. 2020;8(8):CD013359. doi: 10.1002/14651858.CD013359.pub2 32853411 PMC8078611

[19] W.H. Organization. Global tuberculosis report 2021. 2021.

[20] Reitsma JB, Rutjes AWS, Khan KS, Coomarasamy A, Bossuyt PM. A review of solutions for diagnostic accuracy studies with an imperfect or missing reference standard. J Clin Epidemiol. 2009;62(8):797–806. doi: 10.1016/j.jclinepi.2009.02.005 19447581

[21] Schiller I, van Smeden M, Hadgu A, Libman M, Reitsma JB, Dendukuri N. Bias due to composite reference standards in diagnostic accuracy studies. Stat Med. 2016;35(9):1454–70. doi: 10.1002/sim.6803 26555849

[22] Naaktgeboren CA, Bertens LCM, van Smeden M, de Groot JAH, Moons KGM, Reitsma JB. Value of composite reference standards in diagnostic research. BMJ. 2013;347:f5605. doi: 10.1136/bmj.f5605 24162938

[23] Higgins JPT, Thompson SG, Deeks JJ, Altman DG. Measuring inconsistency in meta-analyses. BMJ. 2003;327(7414):557–60. doi: 10.1136/bmj.327.7414.557 12958120 PMC192859

[24] Zhou Y, Dendukuri N. Statistics for quantifying heterogeneity in univariate and bivariate meta-analyses of binary data: the case of meta-analyses of diagnostic accuracy. Stat Med. 2014;33(16):2701–17. doi: 10.1002/sim.6115 24903142

[25] Maynard-Smith L, Larke N, Peters JA, Lawn SD. Diagnostic accuracy of the Xpert MTB/RIF assay for extrapulmonary and pulmonary tuberculosis when testing non-respiratory samples: a systematic review. BMC Infect Dis. 2014;14:709. doi: 10.1186/s12879-014-0709-7 25599808 PMC4298952

[26] Yu G, Zhong F, Ye B, Xu X, Chen D, Shen Y. Diagnostic accuracy of the Xpert MTB/RIF assay for lymph node tuberculosis: a systematic review and meta-analysis. Biomed Res Int. 2019;2019:4878240. doi: 10.1155/2019/4878240 31236407 PMC6545759

[27] Penz E, Boffa J, Roberts DJ, Fisher D, Cooper R, Ronksley PE, et al. Diagnostic accuracy of the Xpert® MTB/RIF assay for extra-pulmonary tuberculosis: a meta-analysis. Int J Tuberculosis Lung Dis. 2015;19:278–84. 10.5588/ijtld.14.0262 25686134

[28] Kohli M, Schiller I, Dendukuri N, Yao M, Dheda K, Denkinger CM, et al. Xpert MTB/RIF Ultra and Xpert MTB/RIF assays for extrapulmonary tuberculosis and rifampicin resistance in adults. Cochrane Database Syst Rev. 2021;1(1):CD012768. doi: 10.1002/14651858.CD012768.pub3 33448348 PMC8078545

[29] Macaskill P, Walter SD, Irwig L, Franco EL. Assessing the gain in diagnostic performance when combining two diagnostic tests. Stat Med. 2002;21(17):2527–46. doi: 10.1002/sim.1227 12205697

[30] de Groot JA, Bossuyt PM, Reitsma JB, Rutjes AW, Dendukuri N, Janssen KJ, et al. Verification problems in diagnostic accuracy studies: consequences and solutions. BMJ (Clinical research ed). 2011;343(d4770):d4770. doi: 10.1136/bmj.d4770 21810869

[31] Wright CA, Hesseling AC, Bamford C, Burgess SM, Warren R, Marais BJ. Fine-needle aspiration biopsy: a first-line diagnostic procedure in paediatric tuberculosis suspects with peripheral lymphadenopathy?. Int J Tuberc Lung Dis. 2009;13(11):1373–9. 19861009

[32] Ghariani A, Jaouadi T, Smaoui S, Mehiri E, Marouane C, Kammoun S, et al. Diagnosis of lymph node tuberculosis using the GeneXpert MTB/RIF in Tunisia. Int J Mycobacteriol. 2015;4(4):270–5. doi: 10.1016/j.ijmyco.2015.05.011 26964807

[33] Pandey S, Congdon J, McInnes B, Pop A, Coulter C. Evaluation of the GeneXpert MTB/RIF assay on extrapulmonary and respiratory samples other than sputum: a low burden country experience. Pathology. 2017;49:70–74. 27913043 10.1016/j.pathol.2016.10.004

[34] Sarfaraz S, Iftikhar S, Memon Y, Zahir N, Hereker FF, Salahuddin N. Histopathological and microbiological findings and diagnostic performance of GeneXpert in clinically suspected tuberculous lymphadenitis. Int J Infect Dis. 2018;76:73–81. doi: 10.1016/j.ijid.2018.08.020 30205158

[35] Sharif N, Ahmed D, Mahmood RT, Qasim Z, Khan SN, Jabbar A, et al. Comparison of different diagnostic modalities for isolation of Mycobacterium Tuberculosis among suspected tuberculous lymphadenitis patients. Braz J Biol. 2021;83:e244311. doi: 10.1590/1519-6984.244311 34431905

[36] Coetzee L, Nicol MP, Jacobson R, Schubert PT, van Helden PD, Warren RM, et al. Rapid diagnosis of pediatric mycobacterial lymphadenitis using fine needle aspiration biopsy. Pediatr Infect Dis J. 2014;33(9):893–6. doi: 10.1097/INF.0000000000000312 25361020

[37] Nataraj G, Kanade S, Mehta P. Xpert(®) MTB/RIF for improved case detection of extra-pulmonary TB in a tertiary care setting in urban India. Int J Tuberc Lung Dis. 2016;20(7):890–4. doi: 10.5588/ijtld.15.0849 27287640

[38] Raja R, Sreeramulu PN, Dave P, Srinivasan D. GeneXpert assay - A cutting-edge tool for rapid tissue diagnosis of tuberculous lymphadenitis. J Clin Tuberc Other Mycobact Dis. 2020;21:100204. doi: 10.1016/j.jctube.2020.100204 33305020 PMC7718474

[39] Rammeh S, Ben Rejeb H, M’farrej MK, Znaidi N, Farah F, Ferjaoui M, et al. Cervical node fine needle aspiration: factors influencing the failure rate. Rev Stomatol Chir Maxillofac Chir Orale. 2014;115(2):85–7. doi: 10.1016/j.revsto.2014.02.002 24656861

[40] Robitschek J, Straub M, Wirtz E, Klem C, Sniezek J. Diagnostic efficacy of surgeon-performed ultrasound-guided fine needle aspiration: a randomized controlled trial. Otolaryngol Head Neck Surg. 2010;142(3):306–9. doi: 10.1016/j.otohns.2009.11.011 20172371

[41] Mondoni M, Sotgiu G. Optimizing the endoscopic diagnosis of mediastinal lymphadenopathy: a glimpse on cryobiopsy. BMC Pulm Med. 2022;22(1):355. 36123592 10.1186/s12890-022-02160-2 PMC9487110

[42] Chhajed PN, Vaidya PJ, Mandovra NP, Chavhan VB, Lele TT, Nair R, et al. EBUS-TBNA in the rapid microbiological diagnosis of drug-resistant mediastinal tuberculous lymphadenopathy. ERJ Open Res. 2019;5(4):00008–2019. doi: 10.1183/23120541.00008-2019 31754620 PMC6856492

[43] Lee J, Choi SM, Lee C-H, Lee S-M, Yim J-J, Yoo C-G, et al. The additional role of Xpert MTB/RIF in the diagnosis of intrathoracic tuberculous lymphadenitis. J Infect Chemother. 2017;23(6):381–4. doi: 10.1016/j.jiac.2017.03.001 28372894

[44] WHO Guidelines Approved by the Guidelines Review Committee. Automated real-time nucleic acid amplification technology for rapid and simultaneous detection of tuberculosis and rifampicin resistance: Xpert MTB/RIF assay for the diagnosis of pulmonary and extrapulmonary TB in adults and children: policy update. Geneva: World Health Organization Copyright © World Health Organization 2013; 2013. 25473701

[45] Liu A, Liu S, Lv K, Zhu Q, Wen J, Li J, et al. Rapid detection of multidrug resistance in tuberculosis using nanopore-based targeted next-generation sequencing: a multicenter, double-blind study. Front Microbiol. 2024;15:1349715. doi: 10.3389/fmicb.2024.1349715 38495513 PMC10940340

[46] Ligthelm LJ, Nicol MP, Hoek KGP, Jacobson R, van Helden PD, Marais BJ, et al. Xpert MTB/RIF for rapid diagnosis of tuberculous lymphadenitis from fine-needle-aspiration biopsy specimens. J Clin Microbiol. 2011;49(11):3967–70. doi: 10.1128/JCM.01310-11 21880965 PMC3209093

[47] Ablanedo-Terrazas Y, Alvarado-de la Barrera C, Hernández-Juan R, Ruiz-Cruz M, Reyes-Terán G. Xpert MTB/RIF for diagnosis of tuberculous cervical lymphadenitis in HIV-infected patients. Laryngoscope. 2014;124(6):1382–5. doi: 10.1002/lary.24478 24166585

[48] Biadglegne F, Tesfaye W, Sack U, Rodloff AC. Tuberculous lymphadenitis in Northern Ethiopia: in a public health and microbiological perspectives. PLoS One. 2013;8(12):e81918. doi: 10.1371/journal.pone.0081918 24349151 PMC3857213

