# Supplementary material for: Exploring the accuracy of the Xpert MTB/RIF assay in detecting lymph node tuberculosis: A systematic review and meta-analysis
Source: PLoS One. 2025 May 7;20(5):e0321507. doi: 10.1371/journal.pone.0321507 (PMC12057916; doi:10.1371/journal.pone.0321507)
Supplement: S1 Fig — (ZIP) [file pone.0321507.s001.zip › supporting information/S10 Fig.pdf]

Meta-regression Number of obs = 22
REML estimate of between-study variance tau2 = .3714
% residual variation due to heterogeneity I-squared_res = 99.80%
Proportion of between-study variance explained Adj R-squared = 13.97%
With Knapp-Hartung modification

lnor	Coef.	Std. Err.	t	P>|t|	[95% Conf.	Interval]
specimentype	-.6921596	.323774	-2.14	0.045	-1.36754	-.0167789
_cons	.3754442	.4164687	0.90	0.378	-.4932942	1.244183

S10 Fig: Meta-regression analysis of specificity of FNA samples and tissue samples using Culture as the gold standard
