# Supplementary material for: Exploring the accuracy of the Xpert MTB/RIF assay in detecting lymph node tuberculosis: A systematic review and meta-analysis
Source: PLoS One. 2025 May 7;20(5):e0321507. doi: 10.1371/journal.pone.0321507 (PMC12057916; doi:10.1371/journal.pone.0321507)
Supplement: S1 Fig — (ZIP) [file pone.0321507.s001.zip › supporting information/S11 Fig.pdf]

Meta-Regression(Inverse Variance weights)

Var	Coeff.	Std. Err.	p - value	RDOR	[95%CI]
Cte.	4.741	0.7863	0.1046	----	----
S	0.297	0.2122	0.3954	----	----
Sample ratio	-0.349	0.3444	0.4958	0.71	(0.01;56.07)

Tau-squared estimate = 0.0000 (Convergence is achieved after 1 iterations)
Restricted Maximum Likelihood estimation (REML)

No. studies = 4
Filter OFF
Add 1/2 to all cells of the studies with zero

(a)

Meta-Regression(Inverse Variance weights)

Var	Coeff.	Std. Err.	p - value	RDOR	[95%CI]
Cte.	4.700	0.9609	0.1284	----	----
S	0.507	0.4268	0.4454	----	----
Decontaminate method	-2.348	3.1282	0.5901	0.10	(0.00;17463586618686200.00)

Tau-squared estimate = 0.4438 (Convergence is achieved after 10 iterations)
Restricted Maximum Likelihood estimation (REML)

No. studies = 4
Filter OFF
Add 1/2 to all cells of the studies with zero

(b)

Meta-Regression(Inverse Variance weights)

Var	Coeff.	Std. Err.	p - value	RDOR	[95%CI]
Cte.	4.700	0.9609	0.1284	----	----
S	0.507	0.4268	0.4454	----	----
homogenization	-2.348	3.1282	0.5901	0.10	(0.00;17463586618686200.00)

Tau-squared estimate = 0.4438 (Convergence is achieved after 10 iterations)
Restricted Maximum Likelihood estimation (REML)

No. studies = 4
Filter OFF
Add 1/2 to all cells of the studies with zero

(c)

Meta-Regression(Inverse Variance weights)

Var	Coeff.	Std. Err.	p - value	RDOR	[95%CI]
Cte.	3.353	0.4303	0.0000	----	----
S	-0.139	0.2143	0.5249	----	----
Sample condition	-0.919	0.6070	0.1509	0.40	(0.11;1.45)

Tau-squared estimate = 1.6116 (Convergence is achieved after 6 iterations)
Restricted Maximum Likelihood estimation (REML)

No. studies = 18
Filter OFF
Add 1/2 to all cells of the studies with zero

(d)

S11 Fig: Results of meta-regression analysis of sample ratio, contaminate method, homogenization and sample condition of FNA samples with CRS as the gold standard: (a) sample rate. (b) purification method. (c) homogenization. (d) sample condition.
