# Supplementary material for: Exploring the accuracy of the Xpert MTB/RIF assay in detecting lymph node tuberculosis: A systematic review and meta-analysis
Source: PLoS One. 2025 May 7;20(5):e0321507. doi: 10.1371/journal.pone.0321507 (PMC12057916; doi:10.1371/journal.pone.0321507)
Supplement: S1 Fig — (ZIP) [file pone.0321507.s001.zip › supporting information/S12 Fig.pdf]

```
------------------------------------------------------------------------------------------
Meta-Regression(Inverse Variance weights)

 Var              Coeff.       Std. Err.      p - value        RDOR           [95%CI]
------------------------------------------------------------------------------------------
Cte.               3.205        2.3411         0.3045          ----            ----
S                 -1.080        1.0230         0.4017          ----            ----
Decontaminate method
                  -0.999        1.2828         0.5177          0.37      (0.00;91.90)

------------------------------------------------------------------------------------------
Tau-squared estimate =  0.0000 (Convergence is achieved after 1 iterations)
Restricted Maximum Likelihood estimation (REML)

No. studies =  5
Filter OFF
Add 1/2 to all cells of the studies with zero
```

(a)

```
------------------------------------------------------------------------------------------
Meta-Regression(Inverse Variance weights)

 Var              Coeff.       Std. Err.      p - value        RDOR           [95%CI]
------------------------------------------------------------------------------------------
Cte.               3.714        2.4629         0.2706          ----            ----
S                 -0.445        1.1383         0.7333          ----            ----
Sample condition
                   1.240        1.5381         0.5046          3.46  (0.00;2587.07)

------------------------------------------------------------------------------------------
Tau-squared estimate =  0.0000 (Convergence is achieved after 1 iterations)
Restricted Maximum Likelihood estimation (REML)

No. studies =  5
Filter OFF
Add 1/2 to all cells of the studies with zero
```

(b)

S12 Fig: Results of the meta-regression analysis of tissue samples with CRS as the gold standard: (a) Purification methods. (b) Sample conditions.
