# Supplementary material for: Exploring the accuracy of the Xpert MTB/RIF assay in detecting lymph node tuberculosis: A systematic review and meta-analysis
Source: PLoS One. 2025 May 7;20(5):e0321507. doi: 10.1371/journal.pone.0321507 (PMC12057916; doi:10.1371/journal.pone.0321507)
Supplement: S1 Fig — (ZIP) [file pone.0321507.s001.zip › supporting information/S13 Fig.pdf]

Meta-Regression(Inverse Variance weights)

Var	Coeff.	Std. Err.	p - value	RDOR	[95%CI]
Cte.	1.997	0.6982	0.0119	----	----
S	-0.193	0.2156	0.3855	----	----
Sample ratio	0.636	0.3499	0.0892	1.89	(0.90;3.98)

Tau-squared estimate = 1.6033 (Convergence is achieved after 6 iterations)
Restricted Maximum Likelihood estimation (REML)

No. studies = 18
Filter OFF
Add 1/2 to all cells of the studies with zero

(a)

Meta-Regression(Inverse Variance weights)

Var	Coeff.	Std. Err.	p - value	RDOR	[95%CI]
Cte.	2.597	0.6235	0.0008	----	----
S	-0.105	0.2304	0.6550	----	----
Decontaminate method	0.817	0.7966	0.3215	2.26	(0.41;12.36)

Tau-squared estimate = 1.9109 (Convergence is achieved after 6 iterations)
Restricted Maximum Likelihood estimation (REML)

No. studies = 18
Filter OFF
Add 1/2 to all cells of the studies with zero

(b)

Meta-Regression(Inverse Variance weights)

Var	Coeff.	Std. Err.	p - value	RDOR	[95%CI]
Cte.	3.208	0.5213	0.0000	----	----
S	-0.178	0.2320	0.4556	----	----
Homogenisation	-0.335	0.8523	0.7002	0.72	(0.12;4.40)

Tau-squared estimate = 2.0620 (Convergence is achieved after 7 iterations)
Restricted Maximum Likelihood estimation (REML)

No. studies = 18
Filter OFF
Add 1/2 to all cells of the studies with zero

(c)

S13 Fig:Results of the meta-regression analysis of the sample ratio, purification method, and homogenization of cultured FNA samples as the gold standard: (a) Sample ratio. (b) decontamination method. (c) Homogenization.
