# Supplementary material for: Exploring the accuracy of the Xpert MTB/RIF assay in detecting lymph node tuberculosis: A systematic review and meta-analysis
Source: PLoS One. 2025 May 7;20(5):e0321507. doi: 10.1371/journal.pone.0321507 (PMC12057916; doi:10.1371/journal.pone.0321507)
Supplement: S1 Fig — (ZIP) [file pone.0321507.s001.zip › supporting information/S2 File.docx]

S2 File: quality assessments employing the revised Quality Assessment of Diagnostic Accuracy Studies (QUADAS-2) tool

Domain 1: patient selection

Risk of bias: could the selection of patients have introduced bias?

| Signaling question 1: was a consecutive or random sample of patients enrolled? | We scored "yes" if the study enrolled a consecutive or random sample of eligible patients, "no" if the study selected patients by convenience, and "unclear" if the study did not report the manner of patient selection or we could not tell. |
| --- | --- |
| Signaling question 2: was a case-control design avoided? | We did not include in the review studies using a case-control design because this study design, especially when used to compare results in severely ill patients versus those in relatively healthy individuals, may lead to overestimation of accuracy in diagnostic studies. We scored "unclear" if we could not tell. |
| Signaling question 3: did the study avoid inappropriate exclusions? | We included original articles where suspected LNTB patients were recruited. We scored "yes" if the study included either fine-needle aspiration specimens or biopsy specimens of LNTB, and the reference standard includes either composite reference standard or culture. We judged "no" if the study included neither fine-needle aspiration specimens nor biopsy specimens, and so did reference standards. We scored "unclear" if we could not tell. |
| Applicability: are there concerns that the included patients and setting do not match the review question? | We scored "low concern" if patients were evaluated at local hospitals or primary care centres. We scored "high concern" if patients were evaluated exclusively as inpatients at tertiary care centres. We scored "unclear concern" if the clinical setting was not reported or if information was insufficient to allow a decision. We also scored “unclear concern” if Xpert testing was done at a reference laboratory and the clinical setting was not reported for the following reason. It was difficult to tell if a given reference laboratory provided services mainly to very sick patients (inpatients in tertiary care) or to all patients, including very sick patients and those with less severe disease (primary, secondary, and tertiary care). |

Domain 2: index test

Risk of bias: could the conduct or interpretation of the index test have introduced bias?

| Signalling question 1: were the index test results interpreted without knowledge of results of the reference standard? | If the index test results first and the gold standard results later, our score is "Y". If the gold standard results first and the index test results after, our score is "N". If both are tested at the same time, our score is "Y". If the order in which the two results appear is not mentioned, our score is "UC". |
| --- | --- |
| Signalling question 2: If a threshold was used, was it pre-specified? | As the threshold is pre-specified in all versions of Xpert, we answered this question "yes" for all studies. |
| Applicability: are there concerns that the index test, its conduct, or its interpretation differ from the review question? | We note that variations in execution of the test might affect accuracy estimates. We judged "low concern" if the test was performed according to WHO standard operating procedures (WHO 2014), or if the index test was performed as recommended by the manufacturer. We scored "high concern" if the test was performed in a way that deviated from these recommendations. We scored "unclear concern" if we could not tell. In studies that evaluated several different types of specimens, we used the following rule: if ≥ 75% of the specimen types were processed per WHO standard operating procedure (SOP) or as per the manufacturer's instructions, we judged "low concern"; if < 50% of the specimen types were processed per WHOSOP or as per the manufacturer's instructions, we scored "high concern"; and if at least 50% to 74% of the specimen types were processed per WHO SOP or as per the manufacturer's instructions, or if we could not tell, we scored "unclear concern". |

Domain 3: reference standard

Risk of bias: could the reference standard, its conduct, or its interpretation have introduced bias?

We considered this domain separately for the reference standard for detection of lymph node tuberculosis and the reference standard for detection of rifampicin resistance.

| Signalling question 1: is the reference standard likely to correctly classify the target condition? | For detection of lymph node tuberculosis, culture is generally considered the best reference standard. However, limitations are associated with culture; bacterial load is usually low in lymph node tuberculosis, leading to a reduction in the sensitivity of culture.  Concerning the conduct of the reference standard (preparation of the specimen for culture), N-acetyl-L-cysteine-sodium hydroxide is routinely used to homogenize, decontaminate, and liquefy non-sterile specimens for TB culture (American Thoracic Society 2000).  However, lymph node aspirates are usually considered sterile, and standards specify, "specimens collected from normally sterile sites may be placed directly into the culture medium” (American Thoracic Society 2000). Overly processing (sterile) specimens with N-acetyl-L-cysteine-sodium hydroxide may lead to a decrease in viable TB bacteria and consequently false-negative cultures. We scored "yes" if studies did not use N-acetyl-L-cysteine-sodium hydroxide for processing specimens and "unclear" if studies used N-acetyl-L-cysteine-sodium hydroxide. We discussed this further under Discussion and Strengths and weaknesses of the review. For detection of rifampicin resistance, culture-based drug susceptibility testing (DST, also called conventional phenotypic method) is considered to be the best reference standard. Lineprobe assays are alsoWHO-recommendedtests for rifampicin resistance. Aswe extracted data only for studies that used culture-based DST or line probe assays (most oN en MTBDRplus), we answered this question "yes" for all studies. |
| --- | --- |
| Signalling question 2: were the reference standard results interpreted without knowledge of results of the index test? | We scored "yes" if the reference test provided an automated result(e.g. MGIT 960), if blinding was explicitly stated, orif it was clear tha tthe reference standard was performed at a separate laboratory and/or was performed by diL erent people. We scored "no" if the study stated that the reference standard result was interpreted with knowledge of the Xpert Ultra or Xpert MTB/RIF test result. We scored "unclear" if we could not tell. |
| Applicability: are there concerns that the target condition as defined by the reference standard does not match the question? | We judged "high concern" if included studies did not speciate mycobacteria isolated in culture, "low concern" if speciation was performed, and "unclear concern" if we could not tell. If the study only used a composite reference standard, we considered applicability low concern. |

Domain 4: flow and timing

Risk of bias: could the patient flow have introduced bias?

Judgements for overall ʽRisk of bias' assessments.

• If we answered all signalling questions for a domain "yes", then we scored risk of bias as "low".

• If we answered all or most signalling questions for a domain "no", then we scored risk of bias as "high".

• If we answered only one signalling question for a domain "no", we discussed further the "risk of bias" judgement.

• If we answered all or most signalling questions for a domain "unclear", then we scored risk of bias as "unclear".

• If we answered only one signalling question for a domain "unclear", we discussed further the "risk of bias" judgement for the doma

| Signalling question 1: was there an appropriate interval between the index test and the reference standard? | In most included studies, we expected that specimens for index test and verification by culture (or a composite reference standard) would be obtained at the same time, when patients were evaluated for presumptive lymph node tuberculosis. However, even if there were a delay of several days between index test and reference standard, tuberculosis is a chronic disease, and we considered misclassification of disease status to be unlikely, as long as treatment was not initiated in the interim. We judged "yes" if the index test and the reference standard were performed at the same time or if the time interval was less than or equal to seven days, "no" if the time interval was greater than seven days, and "unclear" if we could not tell. |
| --- | --- |
| Signalling question 2: did all patients receive the same reference standard? | For the diagnosis of any form of lymph node tuberculosis we answered this question "yes" if all participants in the study or a subset of participants in the study (for whom we extracted data) received the acceptable reference standard either culture or a composite reference standard. Regarding culture, we acknowledge that it is possible that some specimens could undergo solid culture and others liquid culture as the reference standard. This could potentially result in variations in accuracy, but we think the variation would be minimal. |
| Signalling question 3: were all patients included in the analysis? | We determined the answer to this question by comparing the number of patients enrolled with the number of patients included in the 2 × 2 tables. We answered "yes" if the numbers matched and "no" if there were patients enrolled in the study who were not included in the  analysis. We answered "unclear" if we could not tell. |
