# Supplementary material for: Exploring the accuracy of the Xpert MTB/RIF assay in detecting lymph node tuberculosis: A systematic review and meta-analysis
Source: PLoS One. 2025 May 7;20(5):e0321507. doi: 10.1371/journal.pone.0321507 (PMC12057916; doi:10.1371/journal.pone.0321507)
Supplement: S1 Fig — (ZIP) [file pone.0321507.s001.zip › supporting information/S6 File.docx]

***Search Strategy***

*Date: to October 26, 2023*

1. ***PubMed***

**("tuberculosis, lymph node"[MeSH Terms] OR (("lymph nodes"[MeSH Terms] OR ("Lymph"[All Fields] AND "nodes"[All Fields]) OR "lymph nodes"[All Fields] OR ("Lymph"[All Fields] AND "Node"[All Fields]) OR "lymph node"[All Fields]) AND "Tuberculoses"[Title/Abstract]) OR "lymph node tuberculosis"[Title/Abstract] OR (("tuberculosi"[All Fields] OR "Tuberculosis"[MeSH Terms] OR "Tuberculosis"[All Fields] OR "Tuberculoses"[All Fields] OR "tuberculosis s"[All Fields]) AND "lymph node"[Title/Abstract]) OR "lymphadenitis tuberculous"[Title/Abstract] OR "tuberculous lymphadenitis"[Title/Abstract] OR "Scrofula"[Title/Abstract] OR "Scrofulas"[Title/Abstract] OR "mycobacterial cervical lymphadenitis"[Title/Abstract] OR "cervical lymphadenitis mycobacterial"[Title/Abstract] OR "lymphadenitis mycobacterial cervical"[Title/Abstract] OR "cervical tuberculous lymphadenitis"[Title/Abstract] OR (("Lymphadenitis"[MeSH Terms] OR "Lymphadenitis"[All Fields] OR "adenitides"[All Fields] OR "lymphadenitides"[All Fields]) AND "cervical tuberculous"[Title/Abstract]) OR "tuberculous lymphadenitis cervical"[Title/Abstract]) AND ("Xpert"[All Fields] AND "mtb rif"[All Fields]**

Results: 97

1. ***Embase***

#1 'tuberculous lymphadenitis'/exp

#2 ('lymph gland tuberculosis':ti,ab,kw OR 'lymph node tuberculosis':ti,ab,kw) AND 'lymph nodes tuberculosis':ti,ab,kw OR 'lymphadenitis tuberculosa':ti,ab,kw OR 'lymphnode tuberculosis':ti,ab,kw OR 'tuberculosis of the lymph nodes':ti,ab,kw OR 'tuberculosis, lymph node':ti,ab,kw OR 'tuberculous adenitis':ti,ab,kw OR 'tuberculous lymph node':ti,ab,kw OR 'tuberculous lymph nodes':ti,ab,kw OR 'tuberculous lymphadenopathy':ti,ab,kw OR 'tuberculous lymphadenitis':ti,ab,kw

#3 #1 OR #2

#4 'xpert mtb/rif':ti,ab,kw

#3 AND #4

Results: 67

1. ***Cochrane library***

#1 "Xpert" 572

#2 (lymph node tuberculosis) OR "Lymph Node Tuberculoses " OR "Lymphadenitis, Tuberculous " OR " Tuberculoses, Lymph Node" OR " Tuberculous Lymphadenitis" OR "Lymph Node Tuberculosis " OR "Mycobacterial Cervical Lymphadenitis" OR "Cervical Lymphadenitis, Mycobacterial " OR "Scrofula " OR " Tuberculous Lymphadenitis, Cervical" OR " Cervical Tuberculous Lymphadenitis" OR " Scrofulas" OR " Lymphadenitis, Mycobacterial Cervical" OR " Lymphadenitis, Cervical Tuberculous " 140

#3 #1 #2

Results: 16

1. ***Web of science***

**#1 Tuberculosis, Lymph Node (Topic) OR Lymph Node Tuberculoses (Topic) OR Lymph Node Tuberculosis (Topic) OR Tuberculoses, Lymph Node (Topic) OR Lymphadenitis, Tuberculous (Topic) OR Tuberculous Lymphadenitis (Topic) OR Scrofula (Topic) OR Scrofulas (Topic) OR Mycobacterial Cervical Lymphadenitis (Topic) OR Cervical Lymphadenitis, Mycobacterial (Topic) OR Lymphadenitis, Mycobacterial Cervical (Topic) OR Cervical Tuberculous Lymphadenitis (Topic) OR Lymphadenitis, Cervical Tuberculous (Topic) OR Tuberculous Lymphadenitis, Cervical (Topic) and Preprint Citation Index (Exclude – Database)**

**#2 Xpert MTB/RIF (Topic) and Preprint Citation Index (Exclude – Database)**

**#1 AND #2 and Preprint Citation Index (Exclude – Database)**

**Result: 185**

1. ***Socups***

**TITLE-ABS-KEY ( "lymph node tuberculosis" OR "Lymph Node Tuberculoses" OR "Lymph Node Tuberculosis" OR "Tuberculoses, Lymph Node" OR "Lymphadenitis, Tuberculous" OR "Tuberculous Lymphadenitis" OR "Scrofula" OR "Scrofulas" OR "Mycobacterial Cervical Lymphadenitis" OR "Cervical Lymphadenitis, Mycobacterial" OR "Lymphadenitis, Mycobacterial Cervical" OR "Cervical Tuberculous Lymphadenitis" OR "Lymphadenitis, Cervical Tuberculous" OR "Tuberculous Lymphadenitis, Cervical" ) AND ( "XPERT mtb/rif" )**

**Result: 247**
