# Supplementary material for: Exploring the accuracy of the Xpert MTB/RIF assay in detecting lymph node tuberculosis: A systematic review and meta-analysis
Source: PLoS One. 2025 May 7;20(5):e0321507. doi: 10.1371/journal.pone.0321507 (PMC12057916; doi:10.1371/journal.pone.0321507)
Supplement: S1 Fig — (ZIP) [file pone.0321507.s001.zip › supporting information/S7 Fig.pdf]

```
. metareg lnor specimentype, wsse(Selnor) bsest(reml)

Meta-regression                                      Number of obs  =       9
REML estimate of between-study variance              tau2           =  .04019
% residual variation due to heterogeneity            I-squared_res  =  81.23%
Proportion of between-study variance explained       Adj R-squared  =   0.57%
With Knapp-Hartung modification
```

lnor	Coef.	Std. Err.	t	P>|t|	[95% Conf.	Interval]
specimentype	-.1410207	.165726	-0.85	0.423	-.5329005	.2508591
_cons	-.0381917	.2720916	-0.14	0.892	-.6815861	.6052027

S7 Fig：Meta-regression analysis of the sensitivity of FNA samples and tissue samples using CRS as the gold standard.
