# Supplementary material for: Exploring the accuracy of the Xpert MTB/RIF assay in detecting lymph node tuberculosis: A systematic review and meta-analysis
Source: PLoS One. 2025 May 7;20(5):e0321507. doi: 10.1371/journal.pone.0321507 (PMC12057916; doi:10.1371/journal.pone.0321507)
Supplement: S1 Fig — (ZIP) [file pone.0321507.s001.zip › supporting information/S8 Fig.pdf]

```
. metareg lnor specimentype, wsse(Selnor) bsest(reml)
numerical derivatives are approximate
nearby values are missing

Meta-regression                                       Number of obs  =       9
REML estimate of between-study variance               tau2           =       0
% residual variation due to heterogeneity             I-squared_res  =   0.00%
Proportion of between-study variance explained        Adj R-squared  = 100.00%
With Knapp-Hartung modification
```

lnor	Coef.	Std. Err.	t	P>|t|	[95% Conf.	Interval]
specimentype	.0622268	.0242531	2.57	0.037	.0048772	.1195763
_cons	-.1262601	.0471009	-2.68	0.032	-.2376361	-.0148842

S8 Fig: Meta-regression analysis of specificity of FNA samples and tissue samples using CRS as the gold standard
