# Supplementary material for: Exploring the accuracy of the Xpert MTB/RIF assay in detecting lymph node tuberculosis: A systematic review and meta-analysis
Source: PLoS One. 2025 May 7;20(5):e0321507. doi: 10.1371/journal.pone.0321507 (PMC12057916; doi:10.1371/journal.pone.0321507)
Supplement: S1 Fig — (ZIP) [file pone.0321507.s001.zip › supporting information/S9 Fig.pdf]

```
. metareg lnor specimentype, wsse(Selnor) bsest(reml)

Meta-regression                                      Number of obs  =       24
REML estimate of between-study variance              tau2           =    .8604
% residual variation due to heterogeneity            I-squared_res  =   99.42%
Proportion of between-study variance explained       Adj R-squared  =    1.04%
With Knapp-Hartung modification
```

| lnor | Coef. | Std. Err. | t | P>\|t\| | [95% Conf. | Interval] |
|---|---|---|---|---|---|---|
| specimentype | -.4210955 | .3892918 | -1.08 | 0.291 | -1.228437 | .3862463 |
| _cons | .16317 | .5827138 | 0.28 | 0.782 | -1.045304 | 1.371644 |

S9 Fig: Meta-regression analysis of the sensitivity of FNA samples and tissue samples using Culture as the gold standard
