# Supplementary figures and images for: Exploring the accuracy of the Xpert MTB/RIF assay in detecting lymph node tuberculosis: A systematic review and meta-analysis
Source: PLoS One. 2025 May 7;20(5):e0321507. doi: 10.1371/journal.pone.0321507 (PMC12057916; doi:10.1371/journal.pone.0321507)

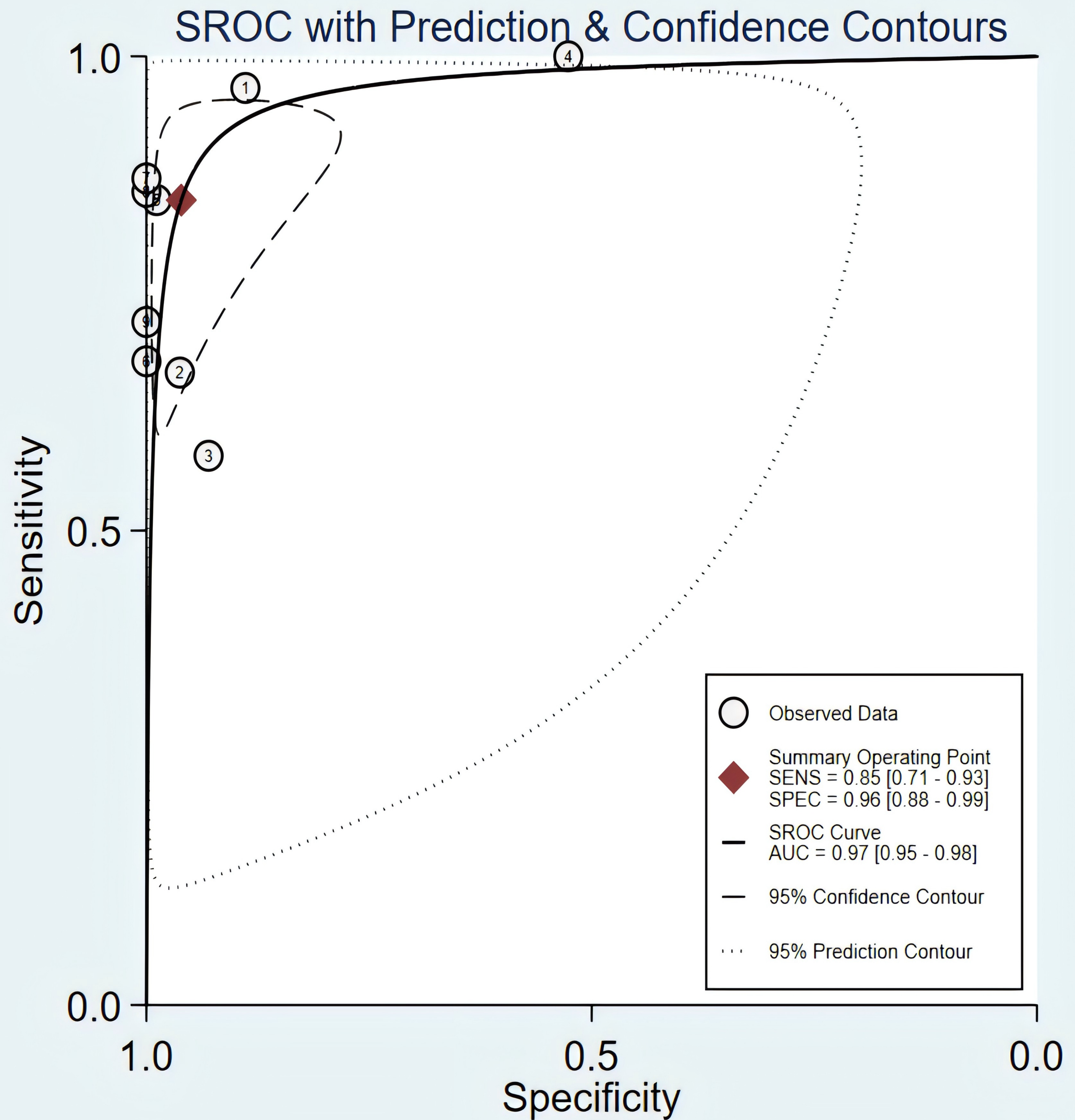

S1 Fig: SROC curves using CRS as the gold standard are included in the articles

Supplement: S1 Fig — (ZIP) [file pone.0321507.s001.zip › supporting information/S1 Fig.pdf]

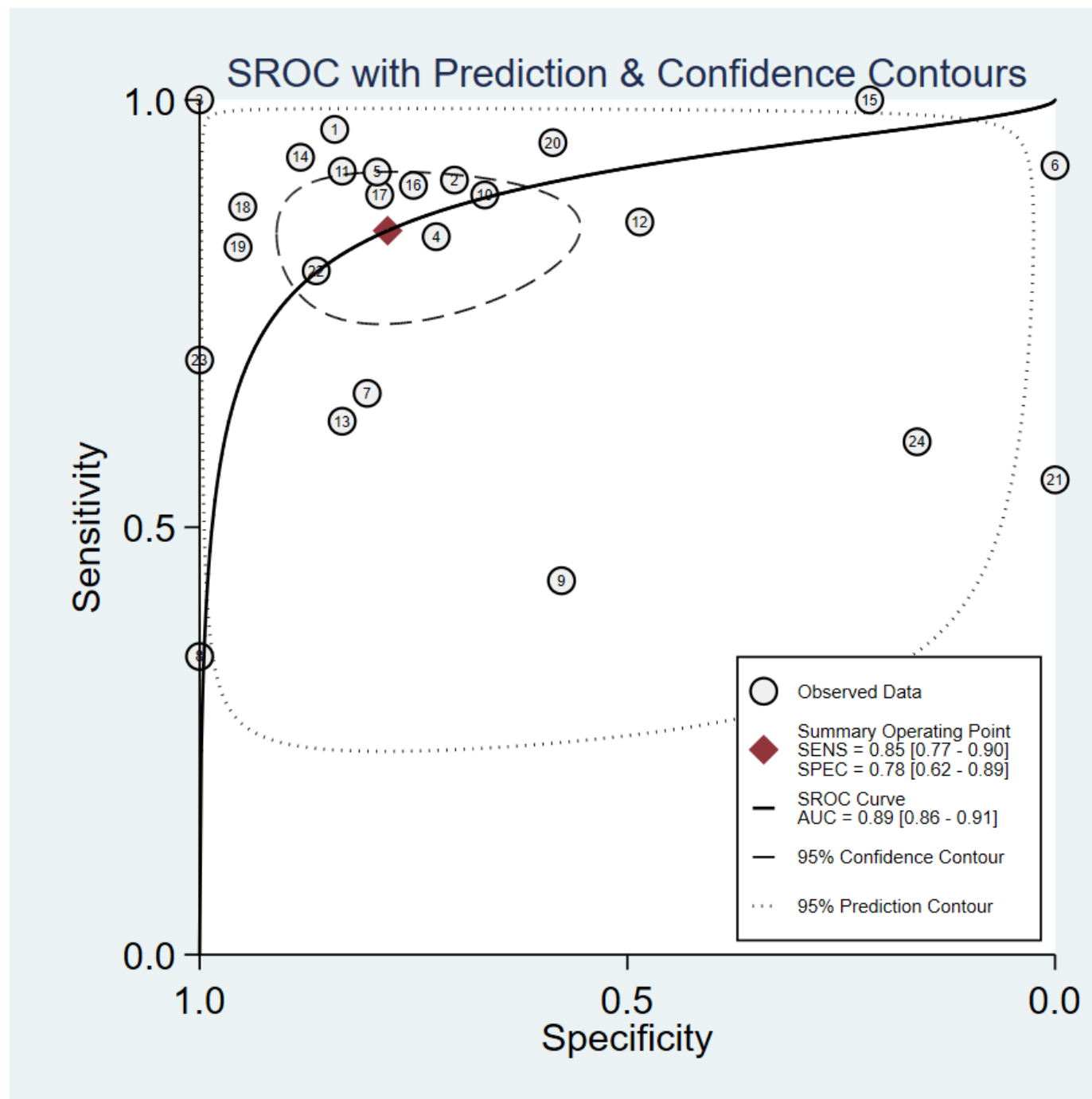

S2 Fig: Forest plots of SROC curves using Culture as the gold standard are included in articles

Supplement: S1 Fig — (ZIP) [file pone.0321507.s001.zip › supporting information/S2 Fig.pdf]

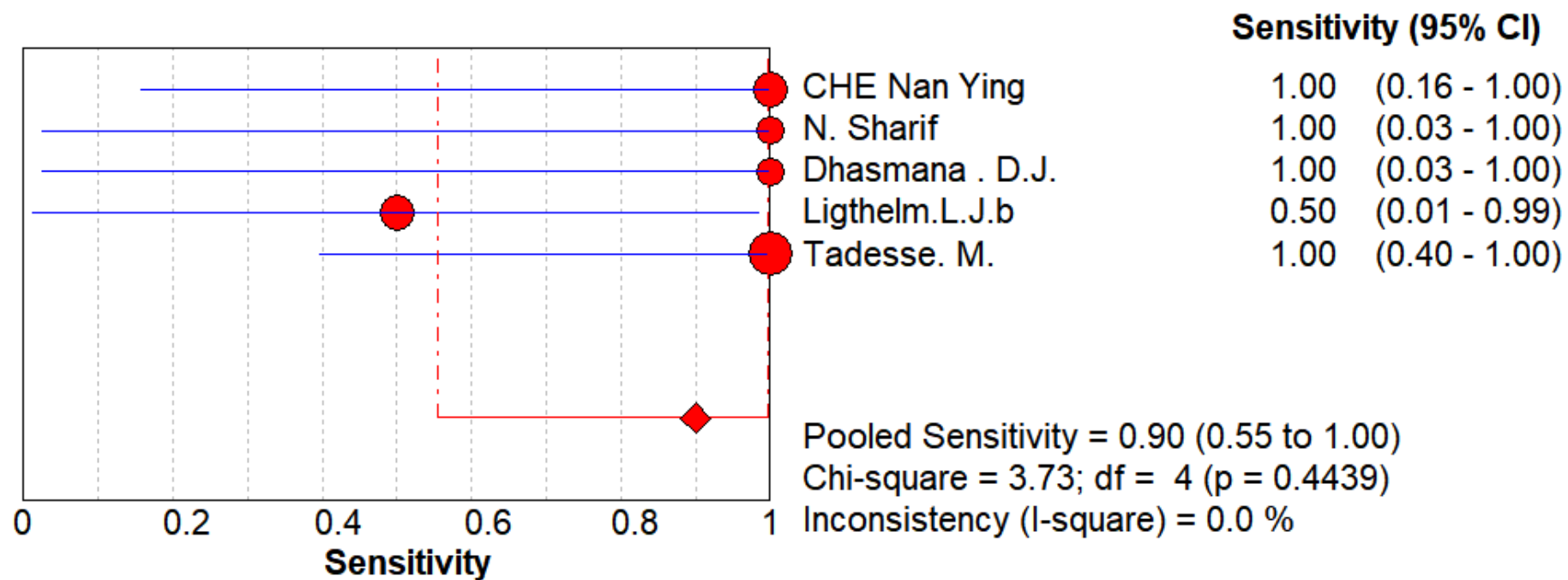

S20 Fig: Plot of sensitivity results for rifampicin resistance.

Supplement: S1 Fig — (ZIP) [file pone.0321507.s001.zip › supporting information/S20 Fig.pdf]

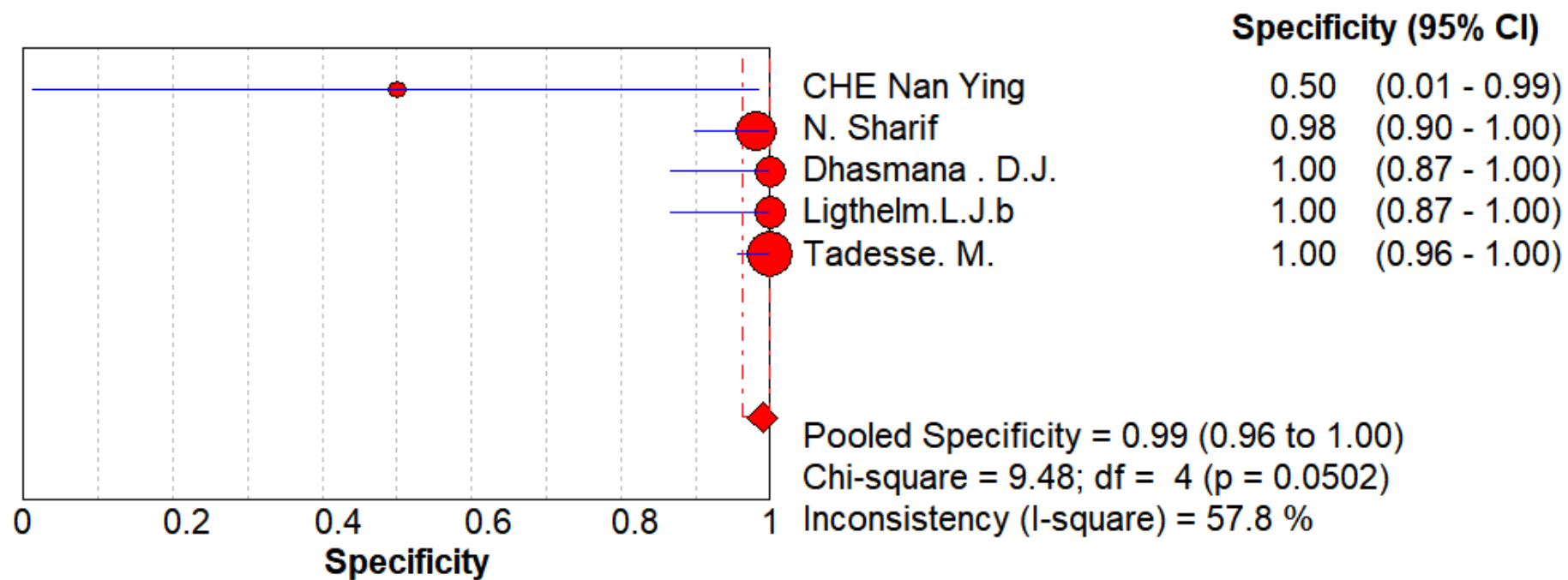

S21 Fig: Plot of specificity results for rifampicin resistance.

Supplement: S1 Fig — (ZIP) [file pone.0321507.s001.zip › supporting information/S21 Fig.pdf]

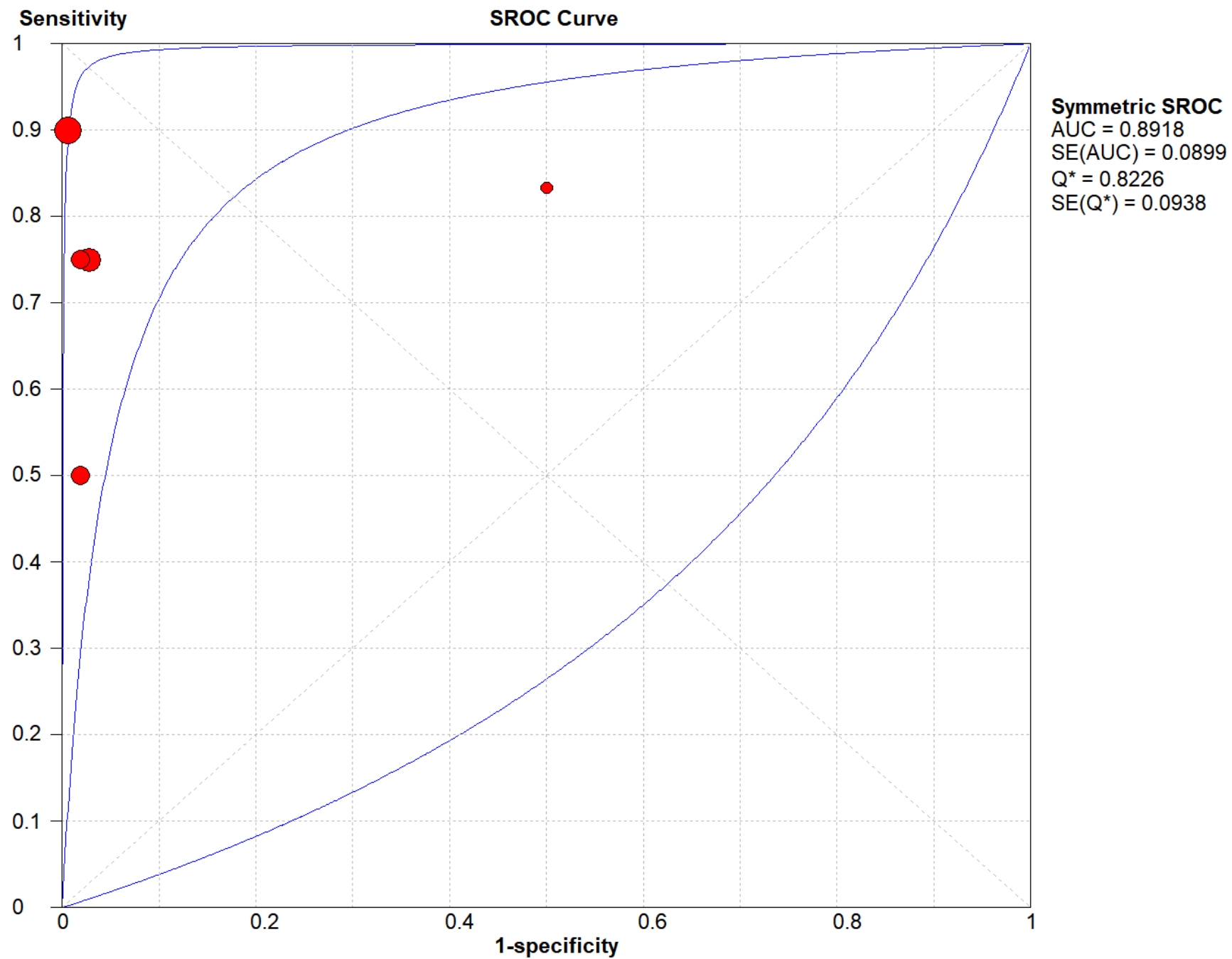

S22 Fig: SROC results for rifampicin resistance.

Supplement: S1 Fig — (ZIP) [file pone.0321507.s001.zip › supporting information/S22 Fig.pdf]

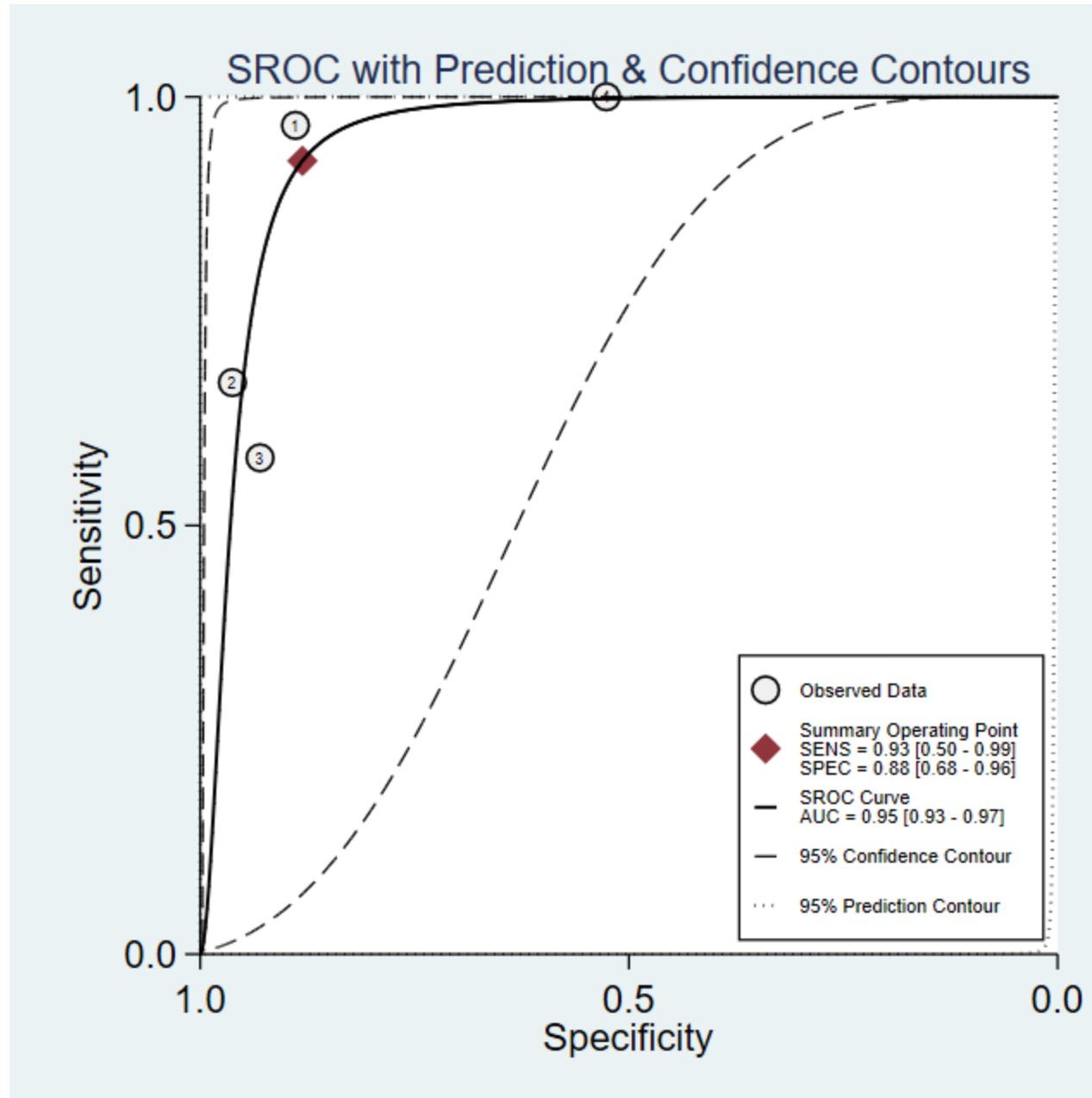

S3 Fig. SROC curve using FNA samples and CRS as the gold standard

Supplement: S1 Fig — (ZIP) [file pone.0321507.s001.zip › supporting information/S3 Fig.pdf]

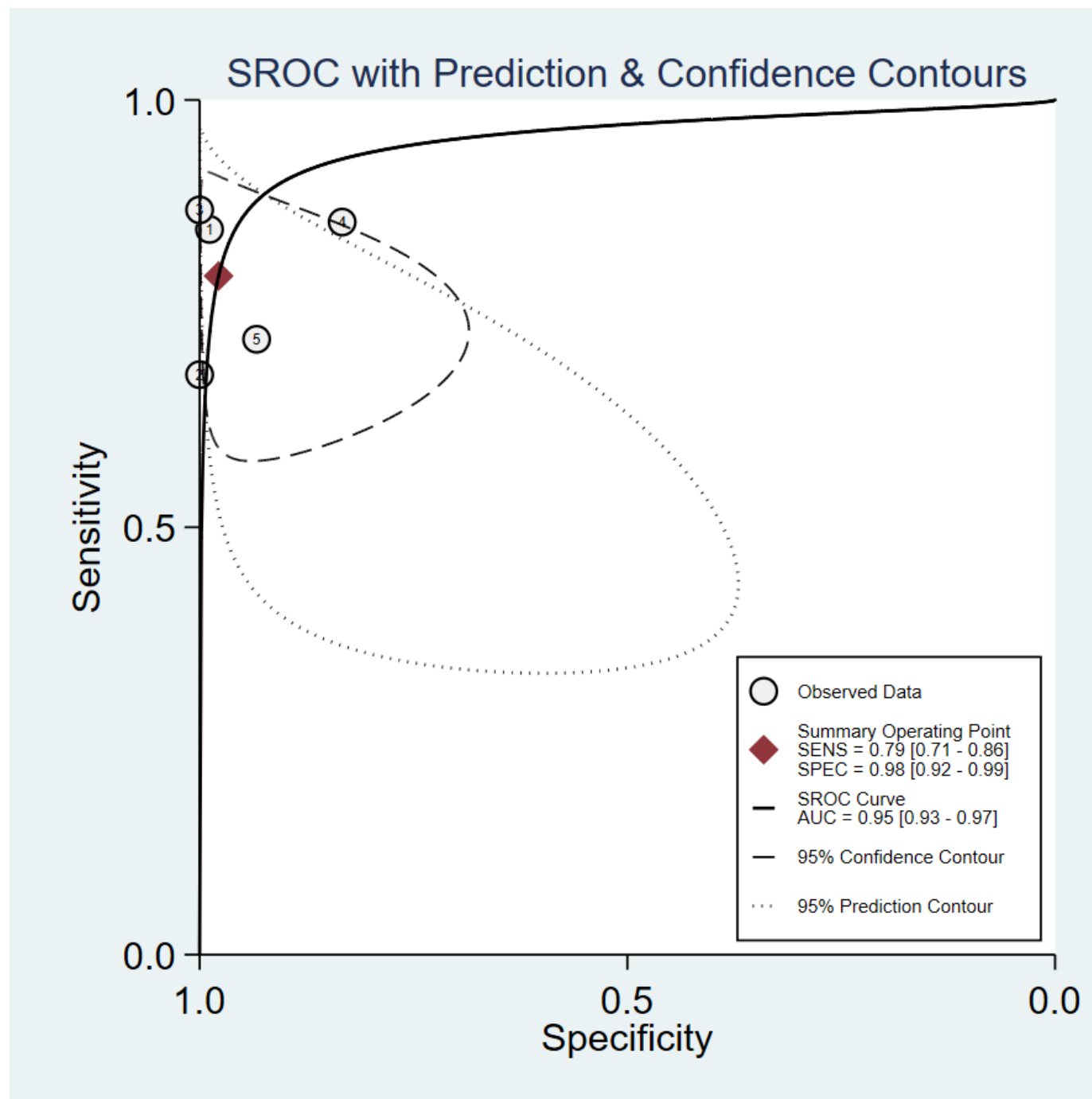

S4 Fig. SROC curve using tissue samples and CRS as the gold standard

Supplement: S1 Fig — (ZIP) [file pone.0321507.s001.zip › supporting information/S4 Fig.pdf]

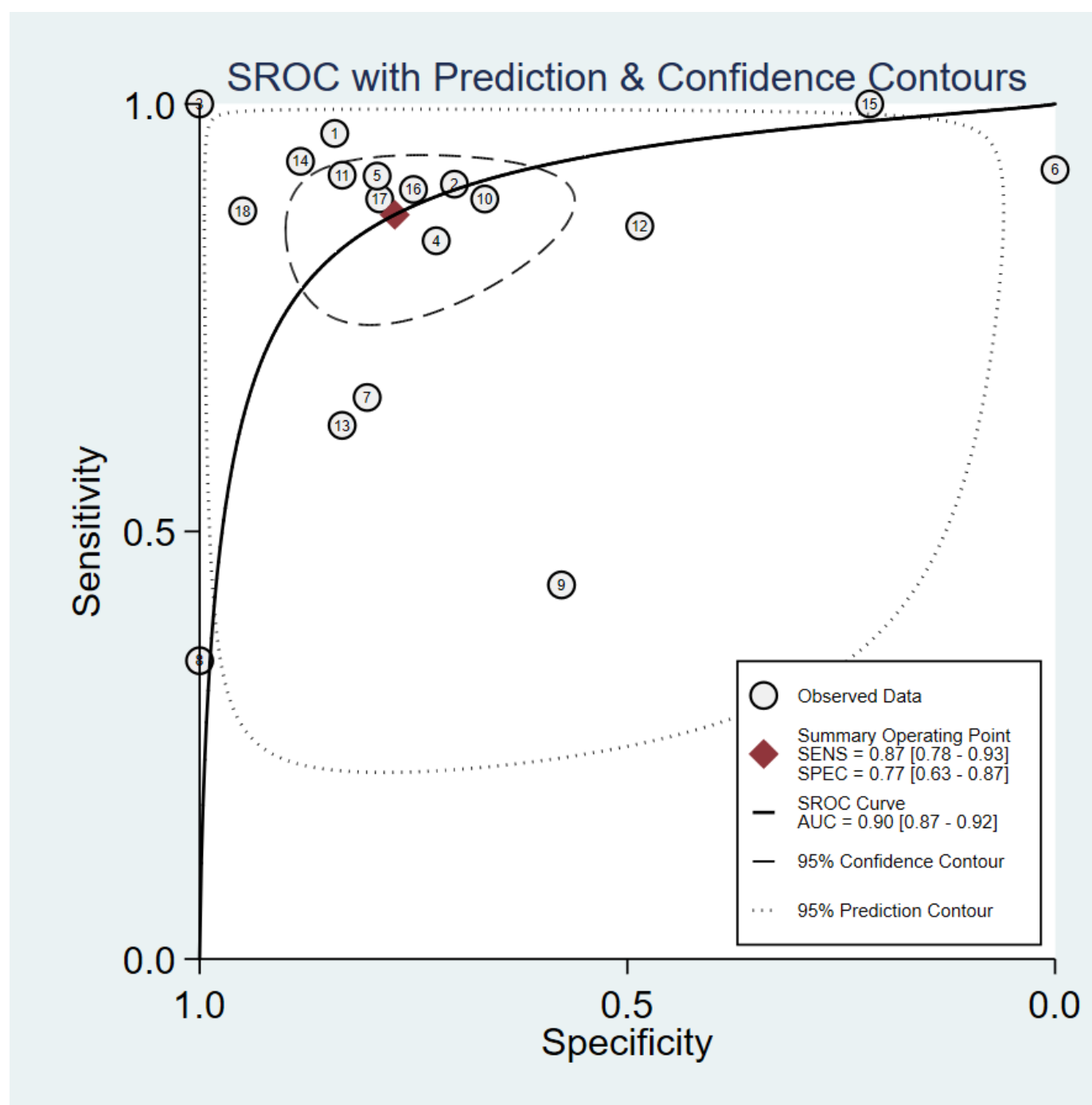

S5 Fig. SROC curve using FNA samples and culture as the gold standard

Supplement: S1 Fig — (ZIP) [file pone.0321507.s001.zip › supporting information/S5 Fig.pdf]

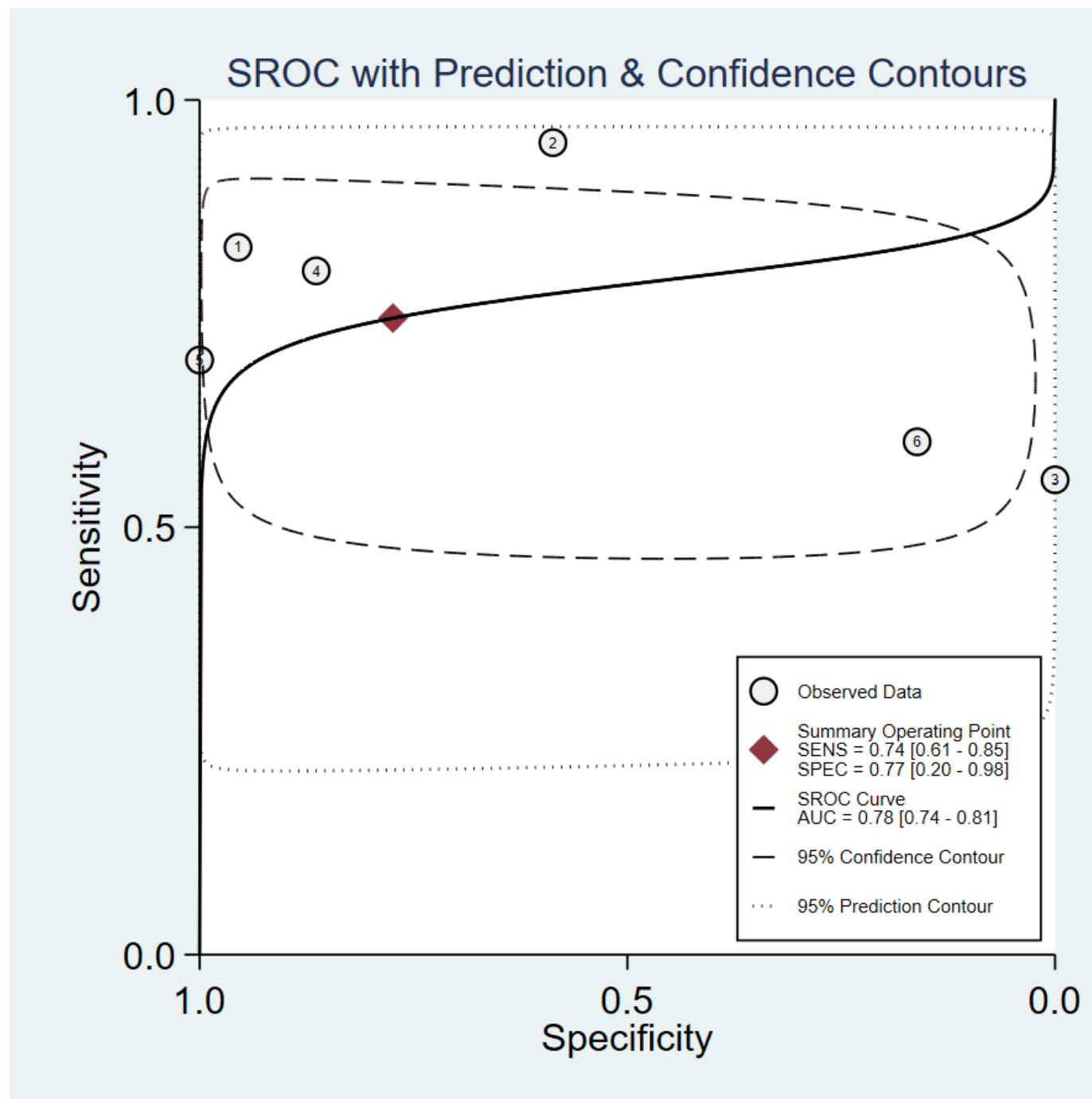

S6 Fig. SROC curve using tissue samples against culture as the gold standard

Supplement: S1 Fig — (ZIP) [file pone.0321507.s001.zip › supporting information/S6 Fig.pdf]
